# p66Shc Protein—Oxidative Stress Sensor or Redox Enzyme: Its Potential Role in Mitochondrial Metabolism of Human Breast Cancer

**DOI:** 10.3390/cancers16193324

**Published:** 2024-09-28

**Authors:** Monika Prill, Vilma A. Sardão, Mateusz Sobczak, Dominika Nowis, Jedrzej Szymanski, Mariusz R. Wieckowski

**Affiliations:** 1 Laboratory of Mitochondrial Biology and Metabolism, Nencki Institute of Experimental Biology PAS, 02-093 Warsaw, Poland; 2 Cellular Immunotherapy Center, Warsaw University of Life Sciences, 02-787 Warsaw, Poland; 3 CNC-Center for Neuroscience and Cell Biology, CIBB—Centre for Innovative Biomedicine and Biotechnology, University of Coimbra, 3004-531 Coimbra, Portugal; vimarisa@ci.uc.pt; 4 Laboratory of Experimental Medicine, Faculty of Medicine, Medial University of Warsaw, 02-091 Warsaw, Polanddominika.nowis@wum.edu.pl (D.N.); 5 Laboratory of Imaging Tissue Structure and Function, Nencki Institute of Experimental Biology PAS, 02-093 Warsaw, Poland; j.szymanski@nencki.edu.pl

**Keywords:** p66Shc, mitochondria, breast cancer, mitochondrial metabolism, oxidative stress

## Abstract

**Simple Summary:**

Mitochondria as peculiar cellular “powerhouses” comprise a source of ATP produced by an oxidative phosphorylation (OXPHOS) system. Additionally, they are implicated in several crucial cellular processes such as regulating the cellular levels of metabolites, Reactive Oxygen Species (ROS) homeostasis, apoptosis, and many others. Thus, it is not surprising that mitochondrial function in cancer cells appears to be a potential promising target in chemotherapy. A substantial amount of evidence indicates that both apoptosis and ROS production could involve a small p66Shc adaptor protein, which can play a dual role as an oxidative stress sensor or “redox” enzyme. The main objective of this work was to determine the role of the p66Shc protein in mitochondrial physiology in MDA-MB-231 breast cancer cells. Thus, the data obtained in our study expanded the knowledge regarding the function of p66Shc protein in cancer cells, indicating the p66Shc protein as a potential therapeutic target for breast cancer treatment.

**Abstract:**

This work presents a comprehensive evaluation of the role of p66Shc protein in mitochondrial physiology in MDA-MB-231 breast cancer cells. The use of human breast cancer cell line MDA-MB-231 and its genetically modified clones (obtained with the use of the CRISPR-Cas9 technique), expressing different levels of p66Shc protein, allowed us to demonstrate how the p66Shc protein affects mitochondrial metabolism of human breast cancer cells. Changes in the level of p66Shc (its overexpression, and overexpressing of its Serine 36-mutated version, as well as the knockout of p66Shc) exert different effects in breast cancer cells. Interestingly, knocking out p66Shc caused significant changes observed mostly in mitochondrial bioenergetic parameters. We have shown that an MDA-MB-231 (which is a strong metastatic type of breast cancer) clone lacking p66Shc protein is characterized by a significant shift in the metabolic phenotype in comparison to other MDA-MB-231 clones. Additionally, this clone is significantly more vulnerable to doxorubicin treatment. We have proved that p66Shc adaptor protein in human breast cancer cells may exert a different role than in noncancerous cells (e.g., fibroblasts).

## 1. Introduction

It is not disputable that functional mitochondria are essential for the proper functioning of cancer cells. Continuous advancements in scientific research have highlighted the intricate network of biological mechanisms and pathways involved in oncogenic transformation as well as the maintenance of the cancerous phenotype. Mitochondrial metabolism in cancer cells significantly deviates from that observed in normal cells. Aerobic glycolysis, the accumulation of Reactive Oxygen Species (ROS), anti-apoptotic signals, and hypoxia are some of the many characteristics typical of cancer cells [1]. To search the metabolic modulators, several therapies targeting the mitochondria of cancer cells have been developed. Nevertheless, this field is rapidly evolving and continues to provide us new insights.

**Figure 1:**
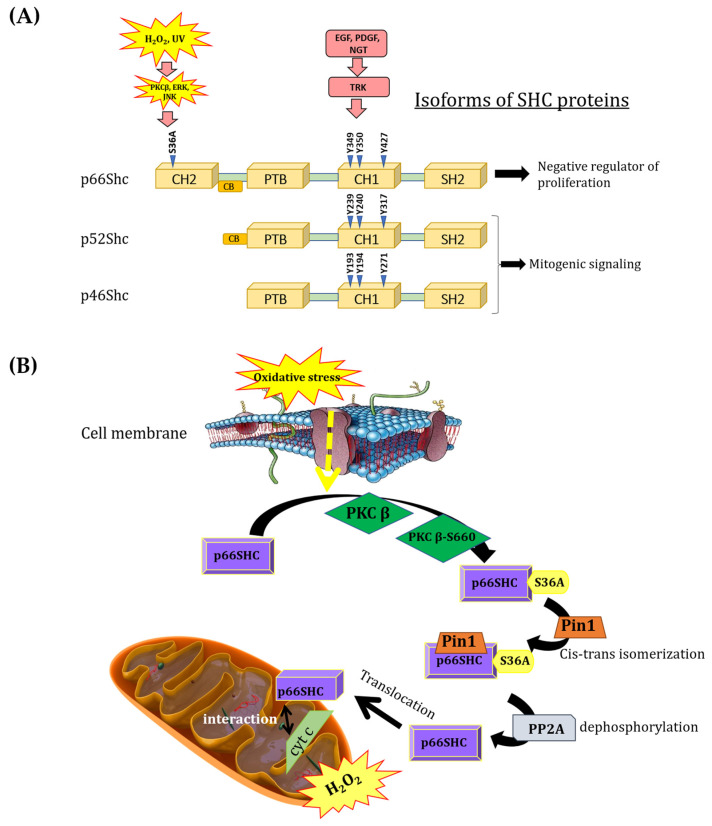
Characteristics of ShcA family protein. (**A**) Domain structure of ShcA isoforms. (**B**) Pro-oxidative signaling pathway of p66Shc isoform. PKCβ—protein kinase Cβ; S660-PKCβ—phosphorylated in S660 form of protein kinase Cβ; Pin1—prolyl isomerase; PP2A—serine/threonine protein phosphatase type 2; cyt. c—cytochrome c.

A significant role of mitochondria in processes related to cell survival and death regulation has been repeatedly demonstrated. p66Shc adaptor protein, which belongs to the ShcA protein family, has been shown as a contributor in ROS production and as the factor influencing processes such as aging and apoptosis. The ShcA family is represented by three different isoforms (p66Shc, p52Shc, p46Shc) encoded by the same gene. All isoforms arise through alternative RNA splicing or alternative translation initiation [2,3,4]. ShcA isoforms are devoid of catalytic activity, exhibiting a unique and highly conserved domain organization (the PTB–CH1–SH2 signature): an N-terminal phosphotyrosine-binding domain (PTB), a central proline-rich domain (CH1), and a carboxy-terminal Src homology 2 (SH2) domain (Figure 1A). In response to growth factor stimuli, p66Shc is phosphorylated at the same tyrosine residues (Y293, Y240, and Y317) as the other two ShcA members (p52Shc and p46Shc) [1,5] but exerts opposite effects, acting as a negative regulator of cell proliferation [6,7,8,9]. Interestingly, p66Shc differs from p52Shc and p46Shc by the presence of an additional N-terminal proline-rich collagen homology domain (CH2), which contains a serine phosphorylation site (S36A) that is critical for its pro-oxidant properties. Moreover, p66Shc contains a functional region (CCB, a cytochrome c-binding region) within the CH2–PTB domains that is responsible for the interaction of p66Shc with cytochrome c [10] (Figure 1A).

Several lines of evidence suggest that the p66Shc protein exhibits various intracellular localizations. Its presence has been detected in the cytoplasm, mitochondria, and endoplasmic reticulum. Associated with the plasma membrane, p66Shc participates in signal transduction, mediating receptor tyrosine kinase signaling (Ras/MAPK signaling), promoting the activation of Rac1 protein (a small G-protein from the Rho family), and triggering NADPH oxidase-dependent ROS production [11]. Additionally, p66Shc has been found in mitochondria-associated membranes (MAMs) as well as plasma membrane-associated membranes (PAMs) [12,13]. In the nucleus, p66Shc can inhibit FOXO transcription factors (Class O of forkhead box transcription factors), thereby regulating the expression of ROS-neutralizing enzymes [14]. The presence of p66Shc has also been suggested in the mitochondrial intermembrane space (IMS), where it may interact with cytochrome c, contributing to ROS production [10].

The p66Shc pro-oxidative pathway is activated, among other stimuli, by oxidative stress, thereby increasing ROS production in mitochondria and initiating apoptosis [10,15]. Consequently, p66Shc is often referred to as a “redox sensor”, displaying specific characteristics of an oxidoreductase [16]. The mechanism by which p66Shc exerts its pro-oxidative effect under physiological conditions by increasing intracellular ROS levels is still under debate. The process of p66Shc translocation to the IMS in response to oxidative stress begins with the activation of protein kinase Cβ (PKCβ) [10,17,18]. PKCβ is activated during oxidative stress and induces the phosphorylation of p66Shc at the unique S36A residue [19]. The phosphorylated p66Shc is then recognized by prolyl isomerase (PIN1) and undergoes cis–trans isomerization. Then, p66Shc is dephosphorylated by serine/threonine protein phosphatase type 2 (PP2A). In this form, p66Shc is translocated to mitochondria. It could interact with the outer mitochondrial membrane at the regions of MAM formation, or it could be translocated to the intermembrane space, where it is involved (due to the presence of the unique CH2 domain) in transferring electrons from cytochrome c to molecular oxygen, leading to the formation of H_2_O_2_ [12] (Figure 1B). Increased H_2_O_2_ production in mitochondria contributes to elevated intracellular H_2_O_2_ levels, leading to the activation of a self-perpetuating loop of ROS production. Giorgio et al. (2005) [10] demonstrated that H_2_O_2_ produced by p66Shc in mitochondria may induce the opening of the mitochondrial permeability transition pore.

Under physiological conditions, ROS levels are tightly controlled; however, in cancer cells, increased ROS production induces a signaling cascade that mediates the maintenance of the oncogenic phenotype. The dual role of ROS in cancer cells is particularly intriguing. ROS stimulate the proliferation and growth of cancer cells, while the modulation of ROS levels is a promising factor to stimulate senescence and apoptosis during anticancer therapy [20,21]. A growing amount of evidence indicates that the levels of p66Shc could be increased in many cancer cell lines. It is worthwhile to note that only p66Shc (in contrast to other Shc isoforms) was found to be upregulated in highly metastatic variants of the human breast cancer cell line MDA-MB-231 [22]. Breast cancers represent a highly heterogeneous group of tumors, differing from one another in their biological characteristics, clinical course, prognosis, and response to treatment [23]. Lewis et al. (2020) [24] hypothesized that the p66Shc protein, along with other metastasis-promoting genes, supports key stages of the metastatic cascade in breast cancer cells. Moreover, Huang et al. (2019) [25] indicated the existence of possible interactions between the p66Shc protein and the transcription factor STAT3 (Signal Transducer and Activator of Transcription). Thus, our understanding of the role of the p66Shc protein in regulating cancer cell metabolism remains incomplete, and comprehending all aspects of this regulation requires extensive additional research.

There are several reports suggesting a potential influence of p66Shc on cancer cell metabolism. In proliferating cells, especially rapidly dividing ones (such as cancer cells), the “energetic” demand is very high and primarily related to cell division and maintaining cellular redox homeostasis. Soliman et al. (2014) [26] demonstrated a significant role of the p66Shc in the metabolic transformations occurring in cancer cells. Their studies suggest that p66Shc mediates (via feedback) the inhibition of not only the response to growth factors but also glucose metabolism in cancer cells. The observed metabolic shift partially depends on the pathway associated with the mammalian target of rapamycin kinase (mTOR) and the S6 kinase (S6K)—the main effector of mTOR [26,27]. A metabolic shift towards a more glycolytic metabolism, often observed in highly proliferative cells, reduces mitochondrial ROS production. This observation confirms that p66Shc acts as an inhibitor of the mTOR kinase pathway, inhibiting mTOR-dependent anabolic metabolism [26,28].

Taking into account different roles that p66Shc plays in the cell, and its possible impact on cellular metabolism (what could influence the response of cancer cells to chemotherapeutics), the aim of this study was to determine the impact of the p66Shc adaptor protein on mitochondrial bioenergetics and energy metabolism of human breast cancer cells. Additionally, we investigated whether modifying the level of p66Shc would have an effect on the response of cancer cells to treatment with a chemotherapeutic agent—doxorubicin. Taking into account the role of p66Shc in the cellular response to oxidative stress, we also focused on determining whether the modification of p66Shc levels in cancer cells will influence ROS levels and the manifestation of oxidative damage. For this purpose, we generated different clones of MDA-MB-231 and MCF-7: (i) clones containing an empty vector (used as a control cell line); (ii) clones overexpressing non-mutated p66Shc; (iii) clones overexpressing Serine 36-mutated p66Shc; and (iv) clones lacking p66Shc protein (KO).

## 2. Materials and Methods

### 2.1. Cells and Cell Culture

Experiments were conducted using human breast cancer cell lines MDA-MB-231, cat. No.: HTB-26 (ATCC, Rockville, MD, USA) and MCF-7, cat. No.: HTB-22 (ATCC, Rockville, MD, USA) as well as, independently, four “clones” of MDA-MB-231 and MCF-7 in which genome editing was performed, resulting in altered levels of the p66Shc protein: (i) an empty vector cell line; (ii) cell line overexpressing p66Shc; (iii) cell line overexpressing mutation in the Serine 36 form of p66Shc; and (iv) knocked out p66Shc cell line (methods used to obtain genetic modifications are described in Section 2.2 and Section 2.3). The description and characteristics of the above-mentioned clones are provided in Table 1. All clones of the MDA-MB-231 cancer cell line were cultured in DMEM with high glucose, 4.5 g/L, and L-glutamine (Lonza, Basel, Switzerland) supplemented with 10% fetal bovine serum (Life Technologies, Grant Island, NY, USA) and 1% antibiotics (penicillin at 10,000 U/mL, streptomycin at 100 mg/mL) (Merck KGaA, Darmstadt, Germany). Cells were maintained at 37 °C in a humidified atmosphere of 5% CO_2_.

### 2.2. Gene Editing—Clustered Regularly Interspaced Short Palindromic Repeats (CRISPR) Technique

To knock out the p66Shc expression in human MDA-MB-231 and MCF-7 breast cancer cell lines, U6gRNA-Cas9-2A-GFP vectors (subsequently referred to as CRISPR plasmids) (Merck KGaA, Darmstadt, Germany) were used, which involves plasmids based on the CRISPR approach. Three independent target sites were designed: (1) TCCGGAGTGGATTGTACTTGGG; (2) GGCTGGCCAACCCGGCTGGGGG; and (3) TGGCTCCCCCTTAGACCCTGGG. An important aspect is that the designed gRNA sequences exhibit complementarity exclusively to the DNA sequences located in the CH2 region, unique only for the p66Shc protein (and is absent in the other SHC isoforms: p46Shc and p52Shc), ensuring that the other proteins encoded by the SHC1 gene remain unaffected. This is significant, because all three ShcA protein isoforms are encoded by the same SHC1 gene, and the p66Shc protein is produced as a result of alternative splicing. The design of the desired oligonucleotide sequences was conducted using computational tools available at the following link, “https://zlab.squarespace.com/guide-design-resources (accessed on 7 December 2015)”, and the designed CRISPR plasmids were purchased from Merck KGaA (Darmstadt, Germany) (custom sequences).

Cells were seeded at a density of 350,000/well on a 6-well plate for 24 h and cotransfected with 1.5 μg U6gRNA-Cas9-2A-GFP vectors (which encoded the previously described target sites) using a jetPRIME (Polyplus Transfection, Illkirch, France) transfection protocol. The transfection success was determined via fluorescent microscopy. After 48 h, the isolation of clonal cell lines by FACS sorting was conducted. Single cells were sorted into 96-well plates (each cell line transfected with different CRISPR plasmids was sorted into 3 independent 96-well plates). After approximately 1 week of cell expansion, the single colonies for “clonal” appearance were assessed. Each clone was passaged after 60% confluence was reached to 24-well plates, followed by 6-well plates. The cell lysates were collected and the p66Shc level was evaluated using the Western Blot technique. The wild type of MDA-MB-231 or MCF-7 human breast cancer cell lines was used as a control to validate the result for obtained knocked out clones.

### 2.3. Overexpression of p66Shc Protein

pcDNA3.1his p66shc (#32574) and pcDNA3.1his p66shc S36A (#32575) plasmids were obtained from Addgene (Watertown, MA, USA). Introduced change in the pcDNA3.1his p66shc S36A plasmid consisted of mutation at the position-36 serine residue of p66Shc carrying a serine-to-alanine mutation. p66shc (hereafter called ↑ p66shc) and p66shc S36A (hereafter called ↑ mut-S36A-p66Shc) encoding cDNA sequences were cloned into the pLVX-IRES-puro vector (Clontech Laboratories, Inc., Mountain View, CA, USA) for subsequent lentiviral expression and verified with DNA sequencing. HEK293T cells were cotransfected with pLVX-p66shc-WT-IRES-puro or pLVX-p66shc-MUT-IRES-puro or empty pLVX-IRES-puro, envelope (pMD2.G), and packaging (psPAX2) vectors using a standard calcium phosphate protocol. pMD2.G and psPAX2 plasmids were obtained from professor Didier Trono (École Polytechnique Fédérale de Lausanne, Switzerland). Lentivirus-containing supernatants were collected 48 h post transfection, centrifuged overnight at 4 °C at 3000× *g*, and added to the culture of MDA-MB-231 cells for 24 h in the presence of 6 µg/mL of polybrene (Merck KGaA, Darmstadt, Germany). A mixture of positive clones was selected with puromycin (Merck KGaA, Darmstadt, Germany) and evaluated with Western Blotting. Wild types of MDA-MB-231 and MCF-7 human breast cancer cell lines were used as controls to validate the result for obtained overexpressed clones.

### 2.4. Proliferation Rate

The proliferation rate of individual MDA-MB-231 clones was assessed using the Guava Muse Cell Analyzer flow cytometer (Merck KGaA, Darmstadt, Germany). The cells were seeded at the density 2.5 × 10^4^/cm^2^. Each clone was seeded in triplicate in a multi-well plate. After 24 h, the cells were detached using trypsin. Subsequently, 20 μL of the cell suspension was added to 380 μL of Muse™ Count & Viability Reagent (Merck KGaA, Darmstadt, Germany), gently vortexed, and incubated for 2 min at room temperature before being analyzed with the Guava Muse Cell Analyzer. The procedure was repeated at 48 h and 72 h after seeding, respectively.

### 2.5. Sample Preparations and Western Blot (WB)

Cells were collected and then lysed in a cold lysis buffer (50 mM Tris, pH 7.5, 150 mM NaCl, 1% Triton, 0.1% SDS, 1% sodium deoxycholate) containing a protease inhibitor cocktail (Merck KGaA, Darmstadt, Germany) and a phosphatase inhibitor cocktail (Merck KGaA, Darmstadt, Germany), added prior to use. The samples were incubated on ice for 30 min and then centrifuged at 10,000× *g* for 20 min at 4 °C to remove insoluble cellular material. The protein concentration in the supernatants was determined using the Bradford method. The samples for SDS-PAGE were denatured in a reducing Laemmli loading buffer at 95 °C or 45 °C (for OXPHOS complex detection) for 5 min. The cell lysates (10–40 μg protein) were separated by SDS-PAGE in 8% or 10% polyacrylamide gels and transferred onto PVDF membranes (Bio-Rad Laboratories, Munich, Germany) for 90 min at 300 mA. The membranes were blocked using an Odyssey Blocking buffer (Li-Cor Bioscience, Lincoln, NE, USA) in a TBS-T buffer (1:1) for 1h. Antibodies used for protein detection are listed in Table 2. The appropriate secondary fluorescent antibodies were labeled with IRdye (1:5000) (Li-Cor Biosciences, Lincoln, NE, USA), all in the Odyssey Blocking buffer in the TBS-T buffer (1:1). The relative levels of the detected protein on the membranes were visualized using an Odyssey Infrared Imaging System (Li-Cor Biosciences, Lincoln, NE, USA) with fluorescent objectives. The fluorescence intensity of the membranes was analyzed using the Image™ Studio software for the Odyssey^®^ v3.021, which is compatible with the Odyssey Infrared Imaging System (Li-Cor Biosciences, Lincoln, NE, USA). The amount of the protein is expressed in relation to Glyceraldehyde 3-phosphate dehydrogenase (GAPDH) (or β-actin) as a reference protein.

**Note:** Due to the technical limitation of transferring the overexpressed p66Shc clones (↑p66Shc and ↑ mut-S36A-p66Shc) material/sample to a neighboring lane (p66Shc KO) during the Western Blot procedure, we decided to perform an analysis of the p66Shc protein level in individual clones of MDA-MB-231 on two separate gels. Each individual gel contained physically the same internal control sample (sample of Ctrl clone) that allowed us to later compare and merge the results from the individual blots.

### 2.6. Detection of p66Shc-P-S36A—Immunoprecipitation (IP)

The cell pellets were resuspended in a cold lysis buffer (50 mM NaCl, 50 mM Tris-HCl, pH 7.55, 0.1% Nonident P40, protease and phosphatase inhibitor cocktail). Antibody-immobilized beads were prepared by incubating ShcA (BD Transduction Laboratories, San Diego, CA, USA) antibodies with 20 μL of Protein G PLUS-Agarose beads (Santa Cruz Biotechnology, Santa Cruz, CA, USA) overnight at 4 °C. The immobilized antibodies were incubated with 500 µg protein for 1 h at 4 °C, and the beads were washed three times (1 min centrifugation; 3300× *g*) at 4 °C with the lysis buffer. The final pellet containing immunoprecipitated ShcA bound to the antibody-immobilized beads was used for the Western Blotting analysis where anti-S36A-P-p66hc antibodies were used for detection.

### 2.7. Measurement of ROS

The detection of ROS levels was performed with the use of fluorescent probe CM-H_2_DCF-DA (Life Technologies, Grant Island, NY, USA) according to the manufacturer’s protocol. In brief, cells grown in 24-well plates were washed twice with PBS to remove the medium and then labeled with 5 μM CM-H_2_DCF-DA in PBS containing 5 mM glucose for 30 min at 37 °C in the dark. The cells were washed twice with PBS, and the fluorescence was recorded using a multi-well plate reader (Infinite M200, Tecan, Männedorf, Switzerland) with excitation and emission wavelengths of 585 nm and 520 nm, respectively.

### 2.8. Measurement of Mitochondrial (mt. O_2_^•−^) and Cytosolic (cyt. O_2_^•−^) Superoxide Levels

Cells grown in 24-well plates were incubated for 10 min at 37 °C in the presence of 5 μM MitoSOX (Life Technologies, Grant Island, NY, USA) in PBS containing 5 mM glucose (mt. O_2_^•−^) and for 20 min at 37 °C in the presence of 0.5 μM dihydroethidium (DHE, Life Technologies, Grant Island, NY, USA) in a culture medium (cyt. O_2_^•−^) in the dark, respectively. The cells were washed twice with PBS, and the fluorescence was recorded using a multi-well plate reader (Infinite M200, Tecan, Männedorf, Switzerland) with excitation and emission wavelengths of 510 nm and 595 nm for mt. O_2_^•−^ and excitation and emission wavelengths of 535 nm and 635 nm for cyt. O_2_^•−^, respectively.

### 2.9. Quantification of Protein Level by Sulforhodamine B (SRB) Assay

The protein concentration in each well was determined after the measurements to standardize the examined ROS levels as a function of the protein level. This was performed with the use of an SRB assay, as described above. After the measurements of ROS levels, cells were fixed in 1% acetic acid in ice-cold methanol for at least 24 h and the procedure of the assay was followed according to the manufacturer’s protocol.

### 2.10. Measurement of Mitochondrial Membrane Potential (mt. ΔΨ)

Cells grown in 24-well plates were washed twice with PBS to remove the medium and then incubated in the presence of 5 μM JC-1 (Life Technologies, Grant Island, NY, USA) in PBS containing 5 mM glucose for 15 min at 37 °C in the dark. The cells were washed twice with PBS. The green and red fluorescence values were then measured using a multi-well plate reader (Infinite M200, Tecan, Männedorf, Switzerland) at wavelengths of 488 nm (excitation), 527 nm (green emission), and 590 nm (red emission).

### 2.11. Assessment of Doxorubicin Cytotoxic Properties

To determine the desired concentration of DOX (Merck KGaA, Darmstadt, Germany) resulting in a 10% reduction in the proliferation rate of the studied clones of the MDA-MB-231 cell line, a cytotoxicity test using the sulforhodamine B (SRB) dye method (Merck KGaA, Darmstadt, Germany) was performed. The choice of this test was guided by the inability to use “metabolic” tests, e.g., MTT, XTT, or CCK-8, due to changes in the levels of subunits of individual respiratory chain complexes in the MDA-MB-231 clone exhibiting the silenced expression of the gene encoding the p66Shc protein. These tests are primarily based on mitochondrial dehydrogenases activity (e.g., complex II of OXPHOS), so they could not be used in our study. To determine the optimal final concentration of DOX, cells were seeded on 96-well plates at a density of 9000 cells/well. The next day, the cells were treated with various concentrations of DOX in DMSO as a vehicle.

### 2.12. Visualization of the Mitochondrial Network

Cells were seeded on fat-free glass coverslips at the density of 3 × 10^4^ cells in 24-well plates. After 24 h of seeding, the cells were treated with DOX, while the medium in the non-treated cell lines was replaced with a fresh medium. After 72 h of seeding, the cells were incubated for 30 min in the dark at 37 °C with 20 nM Mito Red probe (Merck KGaA, Darmstadt, Germany). The cells were washed three times with a culture medium and fixed with 4% paraformaldehyde (PFA) at pH 7.2 for 15 min in the dark at room temperature. The cells were then washed twice with PBS for 5 min and incubated for 3 min with Hoechst 33342 (Merck KGaA, Darmstadt, Germany) (dilution of 1:10,000) to visualize cell nuclei and washed again with PBS. Microscope slides were prepared through removing the glass coverslip and embedding it in a drop of Glycergel Mounting Medium, Aqueous (Agilent, Santa Clara, CA, USA), on a microscope slide. Images were taken using a Zeiss Spinning Disc confocal microscope (100× magnification) from randomly selected fields. The analysis of mitochondrial morphology was performed using Image J software, v3.5.1 (NIH, Bethesda, MD, USA).

### 2.13. Extracellular Acidification Rate (ECAR) and Oxygen Consumption Rate (OCR)

Glycolytic activity and mitochondrial respiration measurements were performed using a Seahorse XFe96 analyzer of extracellular fluids, and the rates of extracellular acidification and oxygen consumption were measured using a “Glycolytic Rate Assay Kit” (Seahorse Bioscience, cat. No.: 103344-100; Agilent, Santa Clara, CA, USA), respectively. The measurements were made in accordance with the instructions supplied by the XF Glycolytic Rate Assay Kit manufacturers.

### 2.14. Measurement of Oxygen Consumption Using Oxygraph-2k-OROBOROS Oxygen Electrode

The rate of oxygen consumption by mitochondria in cells was measured using an oxygraph (Oxygraph-2k, Oroboros Instruments GmbH, Innsbruck, Austria). Cells were seeded at a density of 42,000/cm^2^ on a ⌀10 cm dish. After 48 h of incubation, the cells were trypsinized, centrifuged, and then resuspended in 250 μL of a fresh culture medium (without fetal bovine serum—FBS). The cells were then counted using a counting chamber (Neubauer, VWR International Sp. z o.o., Gdansk, Poland) under a light microscope and 4 million cells/2 mL were placed in the measurement chamber. The oxygen consumption measurement was conducted at 37 °C in the culture medium without FBS. Seven minutes after adding the cells to the measurement chamber, ATP synthase inhibitor oligomycin (1 μg/1 mL) was added to inhibit the third respiratory state. Subsequently, every 3 min, the uncoupling agent CCCP (carbonyl cyanide m-chlorophenyl hydrazone) (Abcam, Cambridge, UK) (0.1 μM) was added until maximum oxygen consumption was reached. For each clone, the following parameters were measured: the (i) basal respiration; (ii) oxygen consumption associated with ATP synthesis; (iii) proton leak; and (iv) maximal respiration. Oxygen consumption associated with ATP synthesis was calculated as the difference between basal respiration and respiration after the addition of 1 μg/mL oligomycin. Basal respiration when subtracted by the value of oxygen consumption associated with ATP synthesis gives the proton leak across the inner mitochondrial membrane. Maximal respiration was reached in the presence (CCCP titration) of the uncoupling agent CCCP.

### 2.15. Apoptosis Flow Cytometry Analysis

Apoptosis was verified using FITC Annexin V Apoptosis Detection Kit I (BD Biosciences Pharmingen, San Jose, CA, USA) according to the manufacturer’s protocol. The analysis has been performed using BD LSRFortessa Cell Analyzer. The signal from a 488 nm blue laser using 530/30 filters (annexin V) and 575/26 filters (propidium iodide) was collected.

### 2.16. Mass Spectrometry Analysis

A comparative proteomic analysis was performed at the Thermo Fisher Center for Multiplexed Proteomics (Harvard Medical School) in Boston, USA. A statistical analysis and interpretation of the results were carried out independently by us using dedicated software, Taverna Workbench 2.5 and Morpheus “https://software.broadinstitute.org/morpheus (accessed on 7 April 2022)”, in collaboration with the laboratory led by Michał Dąbrowski, PhD (Bioinformatics Laboratory, Nencki Institute of Experimental Biology, Polish Academy of Sciences).

### 2.17. Statistical Analysis

The statistical analysis of the data was performed with the use of Microsoft Office Excel, v2408 (Microsoft Corporation, Washington, DC, USA) followed by the use of GraphPad PRISM 8.0.2 (GraphPad Software, San Diego, CA, USA). Data are presented as the mean of at least three independent replications ± standard deviation (SD). Multiple comparisons were performed using a one-way analysis of variance (ANOVA) or two-way ANOVA followed by appropriate post hoc tests. Results were considered statistically significant at *p* < 0.05. The following notations were used: * *p* < 0.05, ** *p* < 0.01, *** *p* < 0.001.

### 2.18. Ethics

This study performed with the use of genetically modified cells has been performed according to the approval from the Ministry of Environment (RPW/74231/2023).

## 3. Results

### 3.1. Genetic Modifications of the p66Shc Level in MDA-MB-231 Breast Cancer Cell Line

**Figure 2:**
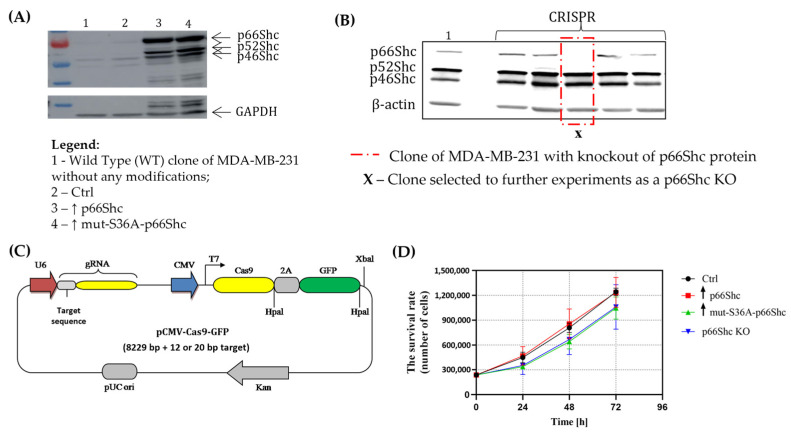
The level of ShcA proteins (p66Shc, p52Shs, and p46Shc) in the MDA-MB-231 clones obtained through (**A**) lentiviral transfection or (**B**) genome editing using the CRISPR/Cas9 method by the Western Blot analysis. (**C**) The schematic of the U6gRNA-Cas9-2A-GFP plasmid, enabling genome editing in MDA-MB-231 cell lines. (**D**) Proliferation rates of the individual MDA-MB-231 clones.

Breast tumors are characterized by an extremely high degree of variability, often resulting from genomic variability [29]. This leads to the development of various breast cancer phenotypes, which may differ not only in their metabolic profile but also in their response to anticancer therapies [30]. The studied human breast cancer cell line—MDA-MB-231—belongs to the basal-like subtype, a more aggressive and metastatic subtype of breast cancer. The p66Shc protein belongs to the ShcA family of adaptor proteins, which includes three isoforms, p66Shc, p52Shc, and p46Shc, all encoded by the same SHC1 gene. However, the p66Shc protein significantly differs from the other isoforms not only in terms of its functional structure but also in the cellular functions it performs. Our use of genetically modified “clones” (variants) of the MDA-MB-231 line, differing in the level of the p66Shc protein, allowed us to elucidate how the p66Shc protein may influence cell physiology, including the metabolism of breast cancer cell mitochondria. The modifications made involved increasing its level (using the lentiviral vector cell transduction technique) (Figure 2A) as well as decreasing it, i.e., obtaining the knockout of p66Shc protein (using the CRISPR-Cas9 technique) (Figure 2B,C). Ultimately, we obtained four independent clones differing in the level of the p66Shc protein, as described in the Section 2. Additionally, it is worth noting that p66Shc levels, as well as other isoforms belonging to the ShcA protein family in the investigated MDA-MB-231 breast cancer cell line (wild type) and the other breast cancer cell line MCF-7, showed their decreased levels in comparison to the control line (MCF-10A—non-tumorigenic normal mammary epithelial cells) (see Supplementary Figure S1).

#### Impact of p66Shc Level Modulation on Proliferation Rate of Genetically Modified Clones of MDA-MB-231

In the first step, we investigated how changes in the level of the p66Shc protein may affect proliferative potential of the examined MDA-MB-231 clones. We have found that increasing the level of the p66Shc protein (↑ p66Shc) does not affect the rate of cell proliferation (Figure 2D). On the other hand, MDA-MB-231 clones exhibiting an elevated level of the mutated form of the p66Shc protein (↑ mut-S36A-p66Shc), as well as those with the knockout of p66Shc protein (p66Shc KO), exhibit lower proliferation rates.

### 3.2. Impact of p66Shc Level Modulation on p66Shc Pro-Oxidative Pathway and ROS Homeostasis

**Figure 3:**
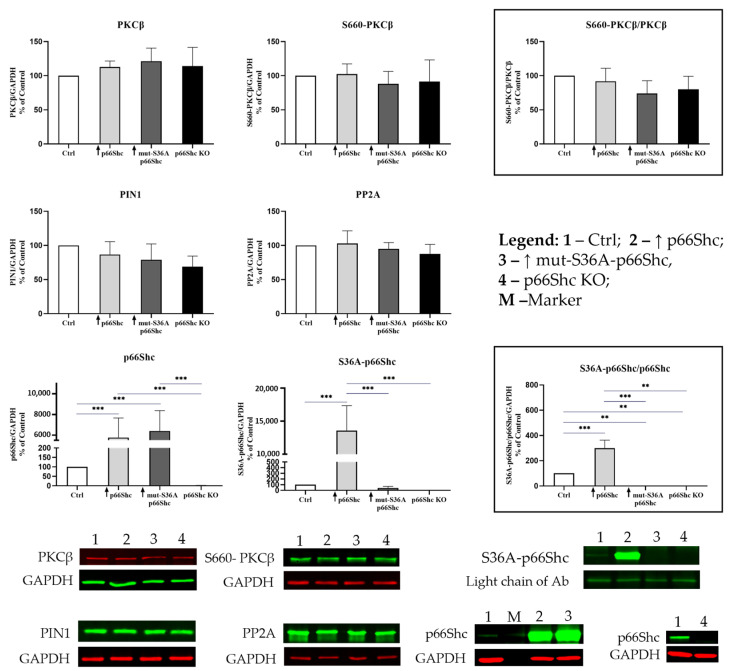
The level of individual proteins involved in the pro-oxidative p66Shc signaling pathway. The levels of proteins were standardized to the GAPDH ± SD. ** *p* < 0.01, *** *p* < 0.001.

In the next step, we have investigated whether changes in the level of p66Shc protein might influence the levels of other proteins involved in the p66Shc pro-oxidative pathway, such as (i) protein kinase Cβ (PKCβ); (ii) its phosphorylated form at the S660 residue (S660-PKCβ); (iii) prolyl isomerase 1 protein (PIN1) responsible for p66Shc isomerization; and (iv) protein phosphatase 2 protein (PP2A) responsible for p66Shc dephosphorylation. The analysis of the p66Shc pro-oxidative signaling pathway in the MDA-MB-231 clones did not reveal significant differences in the levels of PKCβ, PP2A, PIN1, and S36A-p66Shc (Figure 3). 

**Figure 4:**
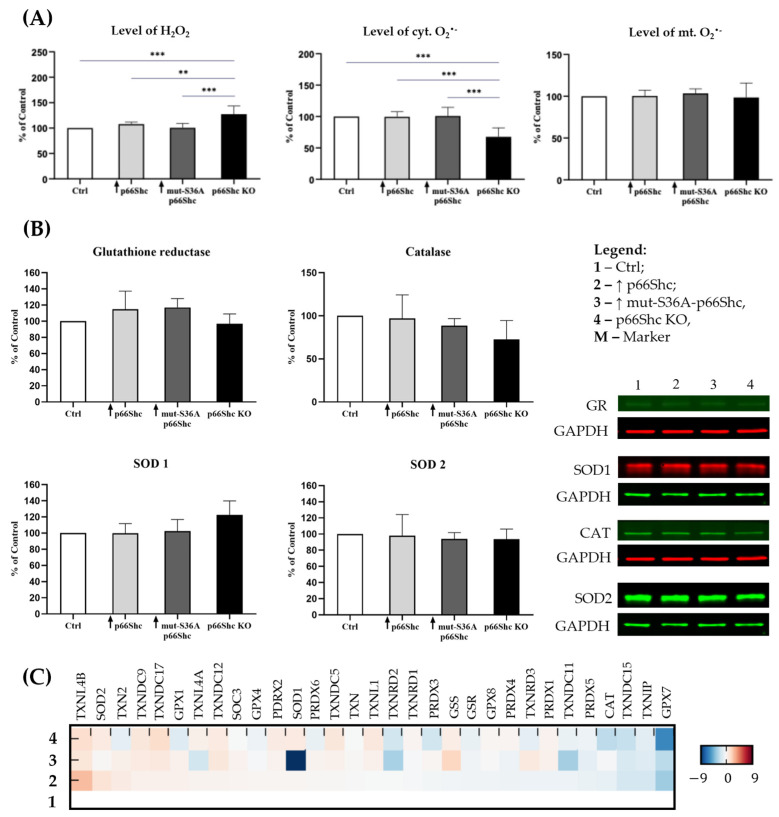
The status of ROS homeostasis. (**A**) The level of ROS species (measured with the use of redox-sensitive fluorophore CM-H_2_DCF-DA, DHE, and MitoSOX, respectively); (**B**) the level of antioxidant enzymes evaluated by WB; the levels of individual antioxidant enzymes were standardized to the GAPDH ± SD; (**C**) the mass spectrometry (MS)–proteomic analysis of antioxidant enzymes in the MDA-MB-231 clones. Samples were standardized to the Ctrl (clone with empty vector). A blue color represents a decrease, while a red color represents an increase in the protein levels; ** *p* < 0.01, *** *p* < 0.001; the levels of hydrogen peroxide (H_2_O_2_) as well as mitochondrial and cytosolic superoxide are shown as a percent of the control value.

The involvement of the p66Shc pro-oxidative pathway in the cellular response to oxidative stress prompted us to investigate whether alterations in the p66Shc level will have an impact on the ROS level in studied MDA-MB-231 clones. Interestingly, the strongest effects have been observed in the knockout of p66Shc protein (p66Shc KO), where the highest level of H_2_O_2_ and the lowest level of cyt.O_2_^•−^ were detected (Figure 4A).

Since the level of ROS largely depends on the efficiency of the antioxidant defense system, we also examined the levels of selected antioxidant enzymes in studied clones characterized by different levels of p66Shc (Figure 4B). Interestingly, we did not observe statistically significant differences in the level of glutathione reductase (GR), catalase (Cat), superoxide dismutase 1 (SOD1), or superoxide dismutase 2 (SOD2), which was also evident in the conducted comparative proteomic analysis (Figure 4C). Additionally, the level of oxidative-damaged proteins also did not differ between investigated clones of the MDA-MB-231 line.

### 3.3. Impact of p66Shc Level Modulation on Cellular Metabolism and Mitochondrial Bioenergetics

The available reports, besides indicating a function related to the cellular response to oxidative stress, also suggest a potential role for the protein p66Shc as a significant regulator of mitochondrial metabolism [10,14,26]. It has been proposed that p66Shc could act as a specific “redox enzyme” with the oxidoreductase activity. By utilizing reducing equivalents from the mitochondrial electron transport chain through the oxidation of cytochrome c, p66Shc contributes to the production of ROS [10,14].

**Figure 5:**
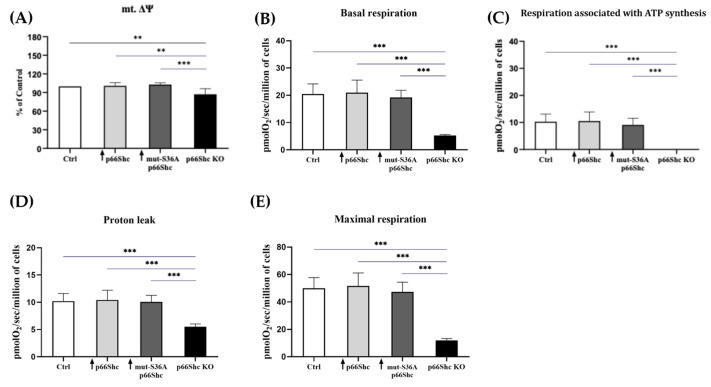
Mitochondrial bioenergetics parameters in the MDA-MB-231 clones. (**A**) Mitochondrial membrane potential and selected functional parameters of the mitochondrial respiratory chain: the (**B**) basal respiration, (**C**) respiration associated with ATP synthesis, (**D**) proton leak, (**E**) maximal respiration in the presence of CCCP. The oxygen consumption rate was calculated as pmolO_2_ per sec per million of cells. ** *p* < 0.01, *** *p* < 0.001.

To investigate how changes in the level of p66Shc protein in the MDA-MB-231 clones might affect their mitochondrial bioenergetics, in the first step, we measured mitochondrial membrane potential. We have found that the knockout of p66Shc protein (p66Shc KO) of the MDA-MB-231 clone has significantly decreased mitochondrial membrane potential in comparison to other MDA-MB-231 clones (ctrl clone, ↑ p66Shc, and ↑ mut-S36A-p66Shc) (Figure 5A).

#### 3.3.1. Mitochondrial Respiration

**Figure 6:**
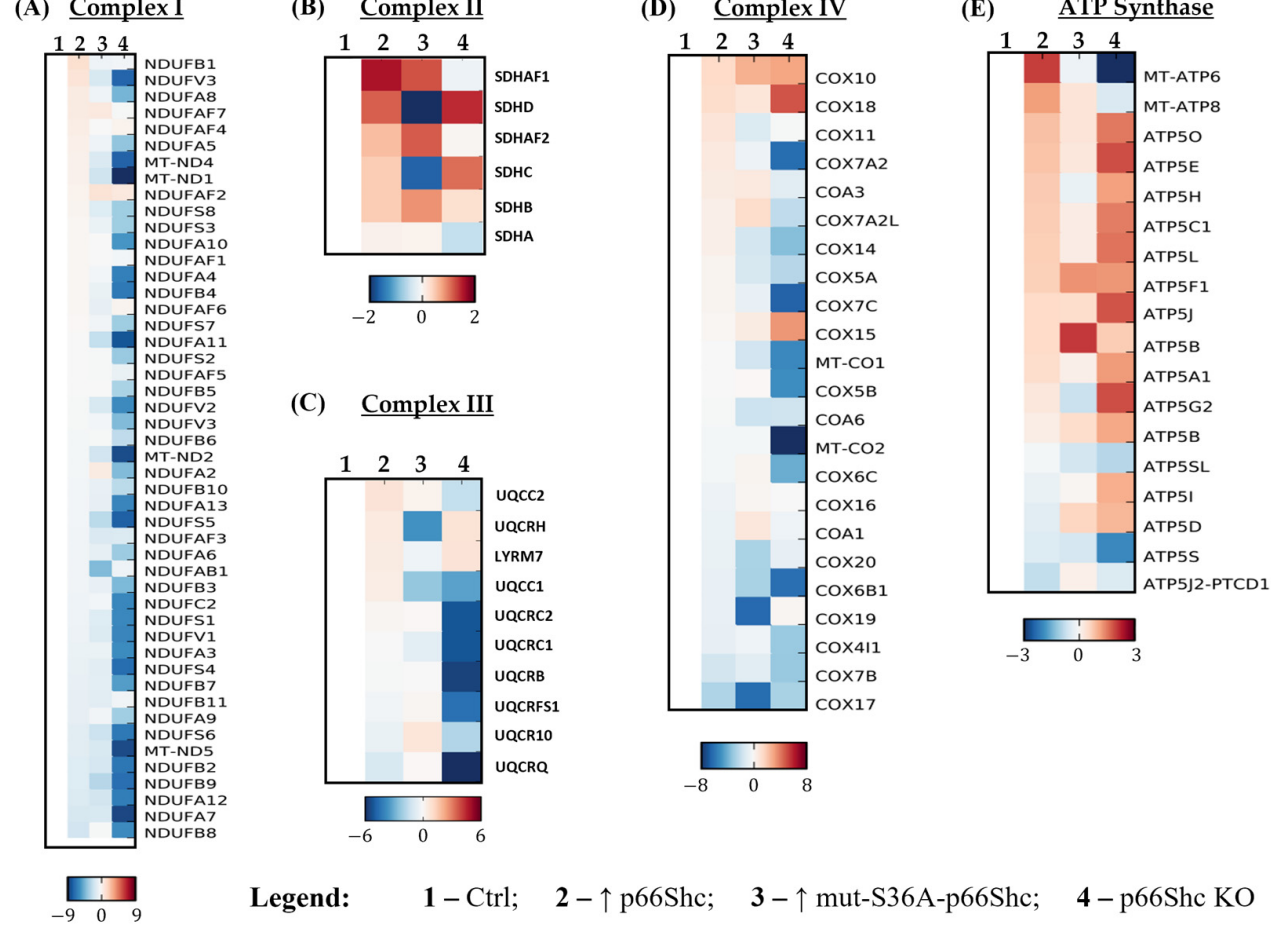
The mass spectrometry (MS)–proteomic analysis of the levels of individual subunits of the OXPHOS in MDA-MB-231 clones. (**A**) Complex I, (**B**) complex II, (**C**) complex III, (**D**) complex IV, and (**E**) ATP synthase. Samples were standardized to the control clone (Ctrl). A blue color represents a decrease, while a red color represents an increase in the protein levels.

Next, to investigate the impact of the p66Shc on mitochondrial function, we examined the mitochondrial respiration rates in the MDA-MB-231 clones. A significant decrease in the basal oxygen consumption rate was observed only in the cells lacking p66Shc protein (p66Shc KO) (Figure 5B). Reduced oxygen consumption at the basal level in the p66Shc KO clone of the MDA-MB-231 line is also accompanied by reduced “leakiness” of the inner mitochondrial membrane (Figure 5D). Moreover, the addition of the oxidative phosphorylation uncoupler, CCCP, revealed that the clone lacking p66Shc protein is characterized by the lowest maximal respiration rate (Figure 5E). Interestingly, similar observations have been made in the investigated parameters for the p66Shc KO clone obtained for another breast cancer cell line, MCF-7 (see Supplementary Figure S2). Additionally, we did not observe a response to oligomycin A treatment in MDA-MB-231 p66Shc KO cells, indicating that there is no respiration linked to ATP synthesis in this clone. No respiration associated with ATP synthesis could be explained by the significantly decreased level of the MT-ATP6 subunit (contributing to proton-transporting ATP synthase activity) in the p66Shc KO clone (see Figure 6E). Moreover, an increase in O_2_ consumption in the response to the CCCP addition suggests that mitochondria in the p66Shc KO clone are at least partially coupled, because we observed appr. a double increase in O_2_ consumption in the response to CCCP addition to the p66Shc KO clone (5.3 ± 0.3 pmolO_2_/sec./1mln. cells and 11.9 ± 1.4 pmolO_2_/sec./1mln. cells; basal and maximal respiration, respectively). 

#### 3.3.2. Mitochondrial Respiratory Chain Composition

Differences in the mitochondrial oxygen consumption could be a result of changes in the level of individual OXPHOS complexes. The comparative proteomic analysis revealed significant differences in the levels of individual subunits of respiratory chain complexes in studied MDA-MB-231 clones. The lowest levels of most detected subunits of complexes I, II, III, and IV were observed in the p66Shc KO clone (Figure 6A–D). On the other hand, most of the subunits of ATP synthase were significantly increased in this clone except MT-ATP6 and ATP5S subunits, which have been found as decreased (Figure 6E). These two subunits of ATP synthase are involved: (i) contributing to proton-transporting ATP synthase activity (MT-ATP6 subunit) and (ii) in the regulation of mitochondrial membrane ATP synthase, necessary for the H^+^ conduction of ATP synthase (ATP5S subunit). Interestingly, the same clone (p66Shc KO) of the MCF-7 line shows a similar relationship in the context of complexes I, II, and III (see Supplementary Figure S3).

#### 3.3.3. Krebs Cycle Enzymes

**Figure 7:**
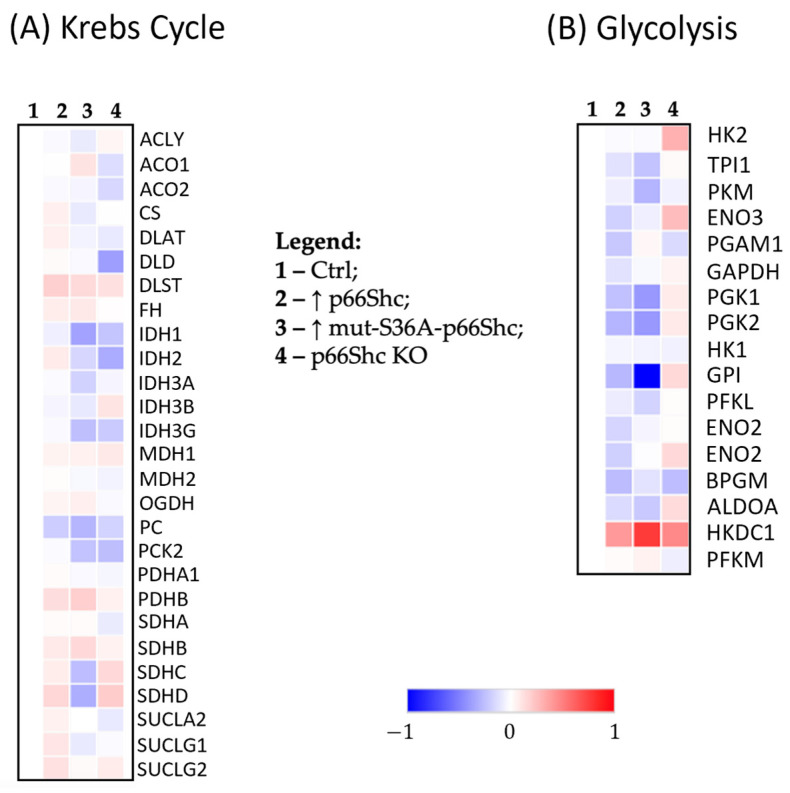
The mass spectrometry (MS)–proteomic analysis of individual enzymes involved in the (**A**) Krebs cycle as well as (**B**) glycolysis determined in clones of the MDA-MB-231. A blue color represents a decrease, while a red color represents an increase in the protein levels.

Additionally, we examined how changes in the level of p66Shc might affect the individual levels of Krebs cycle enzymes. The comparative proteomic analysis revealed differences in the level of individual Krebs cycle enzymes in the investigated MDA-MB-231 clones. We have found that the clone lacking p66Shc and the one with an elevated level of the mutated S36A form of the p66Shc protein are characterized by a decreased level of a significant number of Krebs cycle enzymes (Figure 7A).

#### 3.3.4. Glycolysis Pathway Enzymes

The most visible change is the elevation in the level of the vast majority of glycolytic enzymes in the p66Shc KO clone of the MDA-MB-231 line (Figure 7B). In the case of clones with an elevated level of p66Shc protein (↑ p66Shc and ↑ mut-S36A-p66Shc), we observed exactly the opposite relationship, namely a decrease in the level of most analyzed glycolytic enzymes. It is worth emphasizing that a very similar relationship was also observed for the same p66Shc KO clone of another breast cancer cell line, MCF-7 (see Supplementary Figure S4).

### 3.4. Impact of p66Shc Level Modulation on the Response of Individual Clones of the MDA-MB-231 to the Doxorubicin Treatment

**Figure 8:**
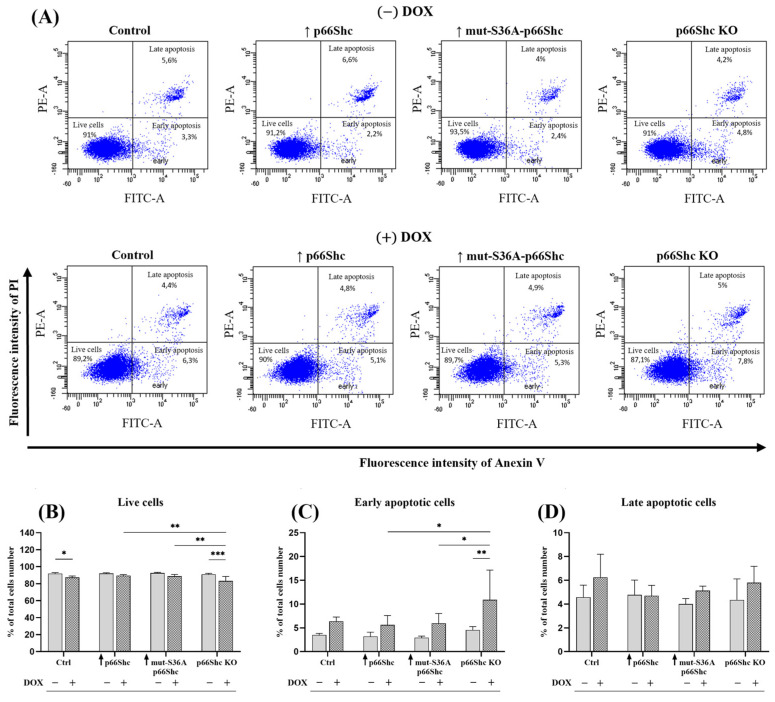
Assessment of cell death (apoptosis) levels in the individual clones (−/+ DOX) of the MDA-MB-231 cell line. (**A**) Representative scatter plots showing changes in annexin V and PI fluorescence intensity. The determination of the percentage of live cells (annexin V−/PI−; lower left quadrant), early apoptotic cells (annexin V+/PI−; lower right quadrant), and late apoptotic cells (annexin V+/PI+; upper right quadrant) in the individual clones of MDA-MB-231 cells (−) DOX and (+) DOX; bar graphs showing the percentage of different cell groups: (**B**) live cells, (**C**) early apoptotic cells, and (**D**) late apoptotic cells; * *p* < 0.05; ** *p* < 0.01; *** *p* < 0.001.

The association of the p66Shc protein with cell proliferation as well as the apoptotic process prompted us to investigate how changes in the level of the p66Shc protein, as well as the response of cells from individual clones of the MDA-MB-231 line to doxorubicin treatment, may affect the apoptotic process in the examined cells, and thus their survival (Figure 8A). The mere change in the level of the p66Shc protein did not significantly affect cell survival. However, it is interesting to note that the change in the level of the p66Shc protein has a significant impact on cell survival when they are treated with doxorubicin (Figure 8B). Specifically, significant differences in the level of live cells were observed mainly between the doxorubicin-treated p66Shc KO clone and the following clones, ↑ p66Shc and ↑ mut-S36A-p66Shc, as shown in Figure 8B. Moreover, statistically significant differences were also observed in the context of the cell response to chemotherapy (Figure 8B,C). Regarding the level of live cells, significant differences concern the following clones treated and untreated with doxorubicin: the control clone (Ctrl) (* *p* < 0.05), and the clone with the knockout of the p66Shc protein (p66Shc KO) (*** *p* < 0.001). In both cases, treatment with DOX resulted in a significant decrease in the number of live cells compared to cells not treated with the chemotherapeutic agent (Figure 8B). Furthermore, the p66Shc KO clone treated with doxorubicin showed a statistically significant increase in the number of early apoptotic cells compared to the same cells but untreated with doxorubicin (Figure 8C). We have not observed changes in the level of late apoptotic cells (Figure 8D).

### 3.5. Impact of the p66Shc Level Modulation on the Doxorubicin Effect on the Mitochondrial Membrane Potential and Mitochondrial Network in MDA-MB-231 Clones 

**Figure 9:**
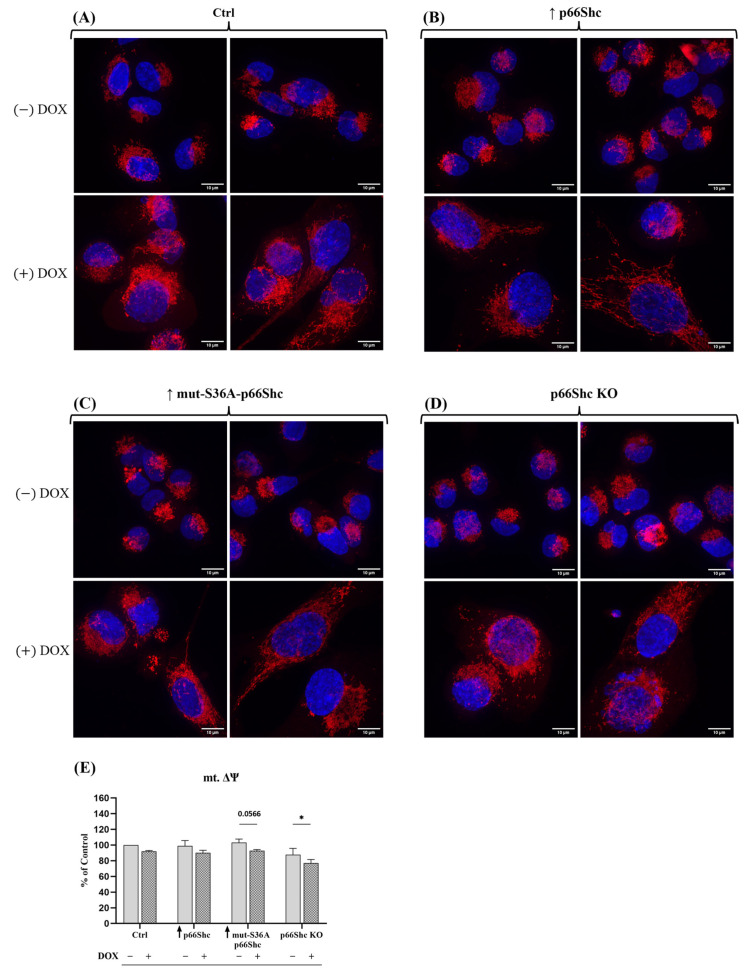
Mitochondrial network morphology in the individual MDA-MB-231 clones treated (+DOX) and untreated (–DOX) with doxorubicin and mitochondrial membrane potential. The (**A**) Ctrl, (**B**) clone with elevated p66Shc protein levels, (**C**) clone with elevated levels of the S36A-mutated form of the p66Shc protein, and (**D**) clone with the knockout of p66Shc; nuclei were stained blue (DAPI) and the mitochondrial network was stained red (MitoRed); (**E**) mitochondrial membrane potential, * *p* < 0.05.

Next, we evaluated whether changes in the level of p66Shc protein influence the mitochondrial network in the individual MDA-MB-231 clones and whether there are differences between the clones in the response to doxorubicin treatment. Treatment of breast cancer cells with doxorubicin induced changes in both the shape of the cell nucleus and the mitochondrial network (Figure 9A–D). The additional analysis of the mitochondrial membrane potential showed a significant impact of doxorubicin treatment mainly in the clone lacking p66Shc protein (Figure 9E), where treatment caused a significant decrease in the mitochondrial membrane potential.

### 3.6. Impact of the p66Shc Level Modulation on the Doxorubicin Effect on the Energetic Profile of the MDA-MB-231 Clones

Taking into consideration the influence of the p66Shc protein on mitochondrial bioenergetic parameters, we were curious whether changes in the p66Shc level could affect the metabolic response of breast cancer cells to doxorubicin treatment. Measurements of ECAR and OCR enabled the determination of the total proton efflux rate (PER)—glycolytic metabolism—and the illustration of the oxygen consumption rates—OXPHOS.

**Figure 10:**
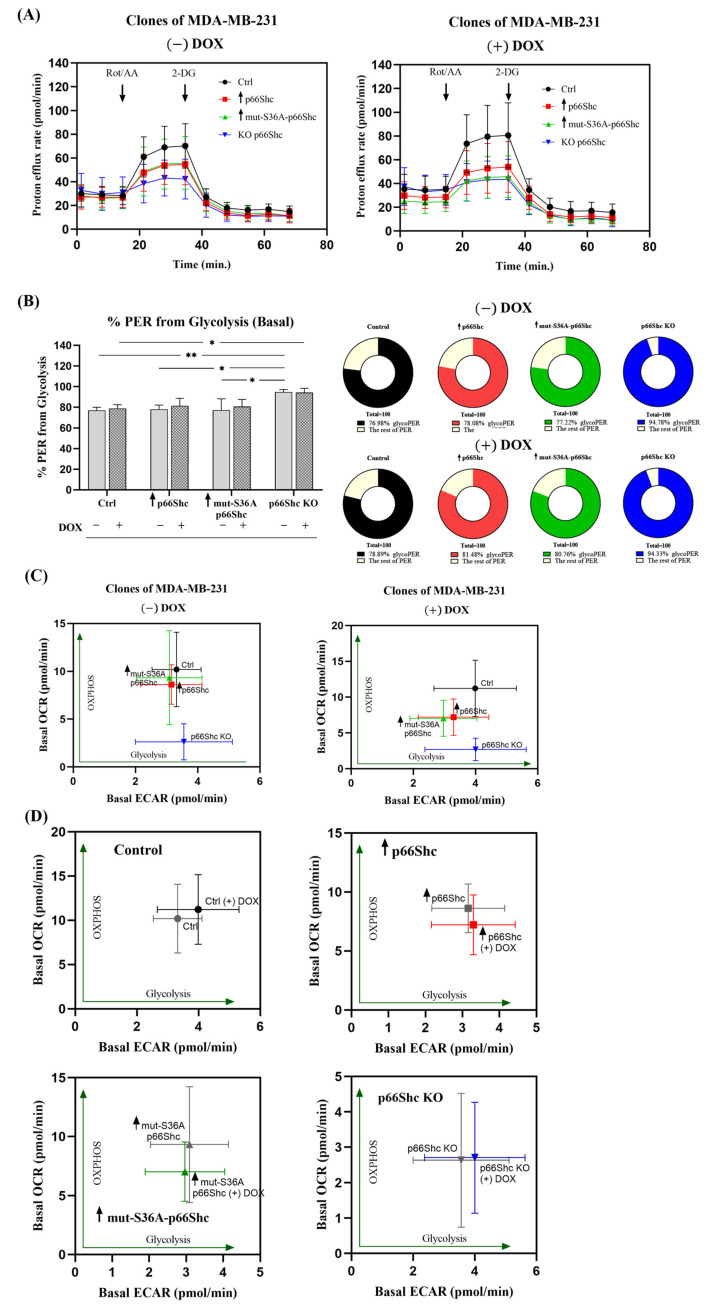
The characteristics of the energetic profile of individual clones of MDA-MB-231 after doxorubicin treatment. (**A**) The proton efflux rate (PER) profile (extracellular acidification profile) for MDA-MB-231 clones untreated and treated with DOX; (**B**) the percentage contribution of “PER from glycolysis” in clones treated and untreated with DOX presented as a bar graph and a pie chart: * *p* < 0.05, ** *p* < 0.01; (**C**) the metabolic phenotype of MDA-MB-231 clones after DOX treatment and (**D**) individual clones (−DOX) and (+DOX), respectively: * *p* < 0.05, ** *p* < 0.01.

To assess the “metabolic signatures” of the examined MDA-MB-231 clones in the response to DOX treatment, we examined “indices” of the two main ATP-generating pathways in the cell, glycolysis and oxidative phosphorylation, which were measured in real-time and expressed as the rate of extracellular acidification (ECAR) and the rate of oxygen consumption (OCR). The parameter representing the percentage contribution of the glycolysis-derived proton efflux rate (PER) allowed for the comparison of examined MDA-MB-231 clones in the light of different levels of the p66Shc protein. We have found that the p66Shc KO clone shows the highest percentage of the “PER from glycolysis” parameter, both before and after DOX treatment (Figure 10A,B). We additionally confirmed a more glycolytic phenotype of the p66Shc lacking clone by the measurement of mitochondrial respiration (Figure 10C). A statistically significant decrease in the rate of basal oxygen consumption was observed in the p66Shc KO clone. Doxorubicin treatment did not induce significant changes in the examined parameters.

**Figure 11:**
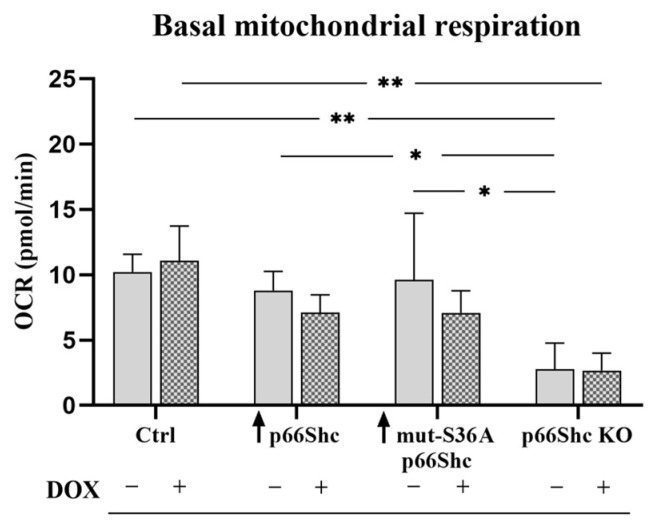
Basal oxygen consumption rate in individual clones of MDA-MB-231 after doxorubicin treatment; * *p* < 0.05, ** *p* < 0.01.

Finally, based on the “basal ECAR” and “basal OCR”, we determined the metabolic phenotype of the examined MDA-MB-231 clones in order to assign them a characteristic “metabolic signature.” Among the examined clones, the most “distinct” (distinctive) is the clone lacking p66Shc protein (p66Shc KO), which clearly exhibits a more glycolytic phenotype (Figure 10D and Figure 11). Interestingly, treatment with doxorubicin resulted in a decrease in cellular respiration in clones ↑ p66Shc and ↑ mut-S36A-p66Shc, while the glycolysis rate remained unchanged (Figure 10D and Figure 11). Interestingly, in the control clone (Ctrl) and the one lacking p66Shc protein (p66Shc KO), treatment with DOX resulted in a shift of the phenotype towards a more glycolytic one (Figure 10D and Figure 11).

## 4. Discussion

Despite being one of the isoforms of the ShcA adaptor protein family, p66Shc is structurally different and contains an additional CH2 domain, through which it influences processes such as ROS production, cell migration, aging, cytoskeleton reorganization, and cell adhesion [16]. Migliaccio et al. (1997) [3] first described the role of p66Shc (as an ShcA isoform) in mitogenic signaling, labeling p66Shc as a negative regulator of cell proliferation. To investigate the impact of p66Shc cellular metabolism, mitochondrial bioenergetics, ROS homeostasis, and the related response of breast cancer cells to treatment with the chemotherapeutic agent doxorubicin, we modulated the level of p66Shc in MDA-MB-231 cancer cells. We generated four “clones” of MDA-MB-231 cells characterized by different levels of the p66Shc protein: the (i) empty vector cell line (as a control); (ii) cell line overexpressing wild type p66Shc; (iii) cell line overexpressing mutation in the S36A form of p66Shc; and (iv) knocked out p66Shc cell line.

Considering the extremely intense cell divisions and hence high proliferation rate typically observed in cancer cells, first, we investigated whether changes in the level of p66Shc could affect the proliferation of the MDA-MB-231 cell line. We have found that the clone lacking p66Shc (p66Shc KO) as well as the clone with overexpressed mutation in the S36A form of p66Shc (↑ mut-S36A-p66Shc) have the lowest proliferation rate compared to the other clones (ctrl and ↑ p66Shc) (Figure 2D). The result obtained for the p66Shc KO clone seems to be in contradiction with the Migliaccio et al. finding [3]; however, a later study by Miller et al. (2019) [31] on prostate cancer cells seems to confirm our observations. They found a 60% decrease in the proliferation of LNCaP-AI cells with reduced levels of p66Shc protein (shRNA p66Shc) compared to control cells (transfected with an empty vector). Furthermore, studies conducted with the use of an MDA-MB-231 cell line highlighted that upon EGF stimulation, p66Shc together with Grb-2 not only enhance proliferation but also promote breast cancer cell migration by activating small GTPases: ARF1 (ADP-ribosylation factor 1) and ARF-6 (ADP-ribosylation factor 6) [32].

The involvement of the p66Shc in the cellular response to oxidative stress prompted us to also investigate how different levels of p66Shc would affect the p66Shc pro-oxidative pathway associated with its phosphorylation at S36A in breast cancer cells. We have demonstrated that the levels of proteins such as PKCβ, its phosphorylated form at S660 residue, PIN1, and PP2A are similar in all MDA-MB-231 clones (Figure 3), indicating that the level of p66Shc has no impact on the level of other proteins from the p66Shc pro-oxidative pathway. Even in the clone overexpressing the wild type of p66Shc protein (↑ p66Shc), where we observe a significant increase in its phosphorylation at the S36A residue, the level of other proteins from this pathway was unchanged. Pinton et al. (2007) [19] demonstrated a clear correlation between the PKCβ-dependent phosphorylation of p66Shc at S36A and early mitochondrial response to oxidative stimuli, but the levels of these proteins are probably sufficient enough to process a higher level of p66Shc involved in the cellular response to oxidative stress via the PKCβ-dependent pathway.

Besides the lack of differences in the level of proteins from the pro-oxidative PKCβ-dependent pathway, we investigated whether alterations in the p66Shc level will have an impact in studied MDA-MB-231 clones on the ROS level. Considering introduced changes in the level of p66Shc, we expected to obtain the highest level of ROS, especially in the clone overexpressing the wild type of p66Shc (↑ p66Shc). Such assumption was confirmed, however, for the clone lacking p66Shc protein and not as expected for the clone overexpressing the wild type of p66Shc (↑ p66Shc) (Figure 4A). Despite the lack of p66Shc protein, the p66Shc KO clone shows the highest level of H_2_O_2_ in comparison to other MDA-MB-231 clones. Conversely, the level of cyt.O_2_^•−^ in this clone is the lowest among all the clones. Our data obtained for the MDA-MB-231 cell line are contradictory to the observations presented by Miller et al. (2019) [31], where LNCaP-AI cells (AI—androgen-independent) lacking p66Shc protein (shRNA p66Shc) had a 40% decreased level of H_2_O_2_. Additionally, an increase in O_2_^•−^ levels in the mitochondria of LNCaP-AI cells overexpressing p66Shc protein has been found under oxidative stress conditions. In the analyzed MDA-MB-231 clones, we did not notice changes in mt.O_2_^•−^ levels (Figure 4A). The discrepancy in the obtained results could be explained by a different type of cancer cell line used in the study and by the fact that the prostate cancer cells are independent of steroid hormones—androgens. It has been demonstrated that p66Shc may exhibit steroid dependence [33,34], which may be relevant to the obtained contradictory results. Extremely interesting and intriguing are the data presented by the group of Miyazawa and Tsuji (2014) [35], who proposed a new role of p66Shc protein as an antioxidant and a protein particularly important in the differentiation of human erythroleukemic K562 cells. According to them, the transcription of p66Shc protein (as the only ShcA isoform) is activated by the antioxidant response element (ARE) pathway (sequences of antioxidant response elements located on some genes) associated with the transcription factor Nrf2 (Nuclear factor erythroid 2-related factor 2). Interestingly, we did not observe statistically significant differences in the level of several antioxidant enzymes as well as in the level of oxidative-damaged proteins in the investigated MDA-MB-231 clones (Figure 4B,C).

Although Otto Warburg suggested that cancer cells have dysfunctional mitochondria and that mitochondrial damage may be one of the causes of cancer, increasingly newer studies on tumor metabolism indicate that functional mitochondria are actually essential for the survival of cancer cells. In this regard, cancer cells do not deactivate mitochondrial energy metabolism but rather change the mitochondrial bioenergetic and biosynthetic state [36]. The triple-negative subtype of breast cancer (TNBC), to which the MDA-MB-231 line belongs, is one of the most aggressive breast cancers, with the worst prognosis and the lowest therapeutic specificity, yet with a higher likelihood of spread and recurrence [30,37]. Interestingly, it has been established that TNBC cells exhibit an 85% overexpression of programmed death-ligand 1 (PD-L1)/programmed cell death protein 1 (PD-1), which is a well-established inhibitory immune checkpoint axis [38]. The effect of immune checkpoint inhibitors is mainly concentrated on improving anti-tumoral immune responses without substantially regulating oncogenic signaling pathways in tumoral cells [38]. In our studies, the level of PD-L1 has not been changed in the clones overexpressing p66Shc or mutated in the S36A form of p66Shc in comparison to the WT MDA-MB-231 (unpublished data) and empty vector-transfected MDA-MB-231 cells (WT MDA-MB-231—99.83%; Ctrl—100%; ↑ p66Shc—100.86%; ↑ mut-S36A-p66Shc—105.17%). Interestingly, the level of PD-L1 seems to be slightly increased in p66Shc KO clones (p66Shc KO—119.83%). Overall, leveraging these technologies could help optimize p66Shc-targeting strategies into precision medicine approaches for TNBC through refined metabolic characterization and multi-modal intervention.

Avagliano et al. (2019) [36] emphasize the glycolytic “character” of the MDA-MB-231 cell line. Several reports suggest that the p66Shc protein could act as a significant regulator of mitochondrial metabolism [10,14,26]. We have found that the knockout of p66Shc protein (p66Shc KO) of the MDA-MB-231 clone has significantly decreased mitochondrial membrane potential in comparison to other MDA-MB-231 clones (Figure 5A). Moreover, our study demonstrated that the MDA-MB-231 clone lacking p66Shc protein has reduced bioenergetic parameters such as the basal respiration and maximal oxygen consumption rate (Figure 5B,E). This could be explained by a decreased level of several crucial subunits from the individual mitochondrial respiratory chain complexes. Interestingly, no respiration associated with ATP synthesis was detected in this clone, which could be a result of decreased levels of MT-ATP6 and ATP5S subunits contributing to proton-transporting of ATP synthase activity. However, an increase in O_2_ consumption in the response to the CCCP addition suggests that mitochondria in the p66Shc KO clone are at least partially coupled. All this may indicate a significant decrease in ATP synthesis efficiency through oxidative phosphorylation in the p66Shc KO clone. Soliman et al. (2014) [26] proposed that p66Shc inhibits glucose metabolism. A lower level of p66Shc led to an increase in glycolysis and induced metabolic transformation [26].

In cancer cells, several changes also occur in the Krebs cycle [39]. Despite previous indications suggesting that cancer cells bypass the TCA cycle and utilize aerobic glycolysis as the main source of ATP, it has been demonstrated that metabolism of some cancer cells may largely depend on the Krebs cycle [39]. We have found that in our generated p66Shc KO MDA-MB-231 clone, the level of several enzymes involved in the Krebs cycle is reduced. Anderson et al. (2018) [39] demonstrated that cancer cells with mutations in the isocitrate dehydrogenase gene (*IDH*) become less sensitive (or insensitive) to treatment with “modified” isocitrate dehydrogenase (IDH) inhibitors. Interestingly, in the MDA-MB-231 clone lacking p66Shc, we observe a decreased level of this protein (Figure 7A). 

Oxygen metabolism largely depends on the efficiency of the mitochondrial respiratory chain. Studies presented by Owens et al. (2011) [40] provide direct evidence of the involvement of dysfunctional oxidative phosphorylation (OXPHOS) complexes in breast cancer development. Moreover, with the use of breast cancer cell lines characterized by different levels of malignancy, including MCF-7 and MDA-MB-231, they demonstrated differences in the expression of genes related to OXPHOS complexes as well as differences in the enzymatic activity of OXPHOS complexes. According to the authors, the activity of complex III was significantly reduced in metastatic and aggressive breast cancer cell lines such as MDA-MB-231 [40]. Our analysis of the OXPHOS profile in the individual clones of the MDA-MB-231 revealed a significant decrease in the level of subunits composing all OXPHOS complexes (I, II, III, and IV) except ATP synthase in the clone lacking p66Shc protein (Figure 6A–E). This could explain observed alterations in the mitochondrial parameters observed for the p66Shc KO clone of MDA-MB-231. Comparing the OXPHOS profiles of aggressive metastatic breast cancer cell line MDA-MB-231 and the less aggressive breast cancer type MCF-7 cell line could suggest a dependence between OXPHOS levels/defects and the malignancy of breast cancer. We have also found that clones of the MCF-7 line (less aggressive breast cancer type) with different levels of p66Shc protein expressed slightly less changes in the OXPHOS proteomic profile in comparison to clones of the MDA-MB-231 line (see Supplementary Figure S3). Indeed, the aggressive metastatic breast cancer cell line—MDA-MB-231—exhibited more aberrations in the OXPHOS activity [40].

The MDA-MB-231 tumor cell line is an estrogen-independent cell line, and triple-negative (ER-, PR-, HER2-), which makes the MDA-MB-231 line an ideal model/tool in experimental studies aimed at developing effective drugs or determining the effect of a particular chemotherapeutic agent. Therapeutic strategies most commonly used for triple-negative breast tumors are mainly based on anthracycline chemotherapeutics [41]. This group includes doxorubicin, which, besides its ability to intercalate between the DNA double-helix bases, also increases the production of free radicals and disrupts mitochondrial bioenergetics, causing mitochondrial oxidative stress [42].

Taking into account the effect of p66Shc protein on metabolic processes, we investigated an association between the level of p66Shc protein and the response to doxorubicin treatment. While changes in the level of p66Shc protein alone did not significantly affect the number of live, early, and late apoptotic cells, treating cells with DOX revealed differences between the MDA-MB-231clones. The greatest changes are observed in the level of live cells and early apoptotic cells (Figure 8B–D). Both the p66Shc KO clone and the control (empty vector) exhibit a significant decrease in cell viability and a pronounced increase in the level of early apoptotic cells in the response to DOX treatment. This may indicate weaker mechanisms protecting cancer cells from initiating the apoptotic process in the control and p66Shc KO clone of the MDA-MB-231 cell line. In contrast, the study performed in immortalized MEF showed that the lack of p66Shc protein is associated with the higher resistance to UV radiation or staurosporine-induced apoptosis [17,43,44]. Moreover, Sakao and Singh (2012) [45] demonstrated in immortalized MEF cells lacking p66Shc an increased resistance to SFN-induced apoptosis and higher resistance to SFN-induced mitochondrial depolarization.

In contrast to our observations, silencing the gene encoding p66Shc protein in MDA-MB-231 and MCF-7 cells contributes favorably to the increased resistance of breast cancer cells to SFN-induced apoptosis [45]. Cañedo et al. (2021) [29] found that in breast cancer cells—Hs578T (classified as triple-negative, TNBC)—showing a high level of p66Shc protein, combined treatment with doxorubicin and a PARP inhibitor caused a 60% increase in the number of apoptotic cells in comparison to cells lacking p66Shc protein. Moreover, this was accompanied by the increase in cytochrome c release only from mitochondria of Hs578T cells characterized by a high p66Shc expression level.

Although the “metabolic phenotypes” of cancer cells still raise many controversies, it is known that mitochondria in cancer cells remain functional. Studies conducted by Koit et al. (2020) [46] additionally indicate a significantly smaller number of mitochondria in MDA-MB-231 cells compared to the MCF-7 line. On the other hand, Reda et al. (2019) [30] showed that mitochondria of MDA-MB-231 cells (despite a more glycolytic profile) were more coupled and had significantly higher respiratory capacity than hormone-responsive MCF-7 cells.

Our analysis of mitochondrial network morphology also indicates a relatively poorly developed mitochondrial network in MDA-MB-231 (Figure 9A–D). Interestingly, the p66Shc KO clone (exhibiting the most glycolytic phenotype among the studied MDA-MB-231 clones) seems to have the least organized mitochondrial network, which may be related to reduced respiratory chain activity in this clone (Figure 5B–D). Additionally, treatment of individual clones with DOX resulted in a significant increase in nuclei size as well as the dispersion of the mitochondrial network around the cell nucleus. We have demonstrated that DOX caused a significant decrease in mt.ΔΨ in the p66Shc KO clone (Figure 9E). Additionally, we assessed the metabolic phenotypes of the generated clones, based on “basal ECAR” and “basal OCR”. We have found that the p66Shc KO clone differs from other clones of MDA-MB-231. It exhibits significantly lower oxygen consumption (Figure 5B–D and Figure 11), thus having the most glycolytic metabolic profile among the studied clones (Figure 10B). This may indicate negligible involvement of oxidative phosphorylation in ATP production in the cells of this clone, which was also confirmed by the lower level of individual OXPHOS subunits (Figure 6). Finally, DOX treatment resulted in a shift of the more glycolytic phenotype of the control clone expressing an empty vector and the clone lacking p66Shc (Figure 10C,D). All these highlight an important role of p66Shac not only in the regulation of cell proliferation and ROS homeostasis but also in the regulation of cellular metabolism via mitochondrial processes.

## 5. Conclusions

The studies presented in this work clearly indicate the influence of the p66Shc protein on the mitochondrial physiology of human breast cancer cells. Nevertheless, the role of p66Shc in the cells of studied MDA-MB-231 clones should be associated with its impact on the mitochondrial metabolism of the cancer cells rather than with the cell’s response to oxidative stress (related to the unique S36A residue presented in p66Shc), as proposed for noncancerous cells (e.g., fibroblasts). We have not demonstrated a link between the elevated level of phosphorylation at S36A, obtained in the clone of MDA-MB-231 with the overexpression of p66Shc protein (as described, for example, in fibroblasts), and increased oxidative stress. This may indicate the presence of a different type of regulation in cancer cells or the existence of additional mechanisms triggered in response to changes in the level of p66Shc and its phosphorylation at S36A.

Interestingly, our results allowed us to identify a previously undescribed relationship between the level of p66Shc protein and the glycolytic metabolic profile of breast cancer cells. The clone of the MDA-MB-231 line (triple-negative breast cancer subtype) with the knockout of p66Shc is characterized by increased levels of many glycolytic parameters (including the level of enzymes involved in glycolysis and the Krebs cycle, the contribution of extracellular acidification PER from glycolysis, and parameters such as basal PER and basal glycolysis) with a simultaneous significant decrease in parameters describing mitochondrial oxygen metabolism. The MDA-MB-231 p66Shc knockout clone exhibits a significant reduction in all functional parameters of the mitochondrial respiratory chain (reduced basal, ATP-linked, and maximal oxygen consumption rates as well as a reduced proton leak). Moreover, the cells of the p66Shc KO clone of the MDA-MB-231 line are definitely more sensitive to treatment with doxorubicin (commonly used in anticancer therapy). Therefore, our data have expanded the fundamental knowledge about the function of the p66Shc protein in cancer cells, which may significantly contribute to the improvement in the latest therapeutic strategies used in the treatment of breast cancer.

## Figures and Tables

**Table 1:** Characteristics of individual genetic modifications performed in clones of MDA-MB-231 and MCF-7 cell lines.

Type of Modification	Description	Abbreviation
Transfection of empty vector	MDA-MB-231 and MCF-7 cell lines transfected with empty vector—control clone	**Ctrl**
Overexpression of p66Shc protein	MDA-MB-231 and MCF-7 cell lines with overexpressed p66Shc level	**↑ p66Shc**
Overexpression of mutated form of p66Shc protein in S36A residue	MDA-MB-231 and MCF-7 cell lines with overexpressed p66Shc level demonstrated mutation in S36A residue	**↑ mut-S36A-p66Shc**
Knockout of p66Shc protein	MDA-MB-231 and MCF-7 cell lines with knockout of p66Shc level	**p66Shc KO**

**Table 2:** List of used primary and secondary antibodies.

Antibody	Host	Company
GAPDH	Rabbit	Abcam, Cambridge, UK
GAPDH	Mouse	Merck KGaA, Darmstadt, Germany
GR	Rabbit	Santa Cruz Biotechnology, Santa Cruz, CA, USA
Catalase	Rabbit	Abcam, Cambridge, UK
OXPHOS	Mouse	Abcam, Cambridge, UK
p66Shc-S36A	Mouse	Merck KGaA, Darmstadt, Germany
PIN1	Rabbit	Merck KGaA, Darmstadt, Germany
PKCβII	Rabbit	Santa Cruz Biotechnology, Santa Cruz, CA, USA
PP2A	Rabbit	Merck KGaA, Darmstadt, Germany
SHC	Mouse	BD Transduction Laboratories, San Diego, CA, USA
SHC	Rabbit	Abcam, Cambridge, UK
SOD1	Rabbit	Santa Cruz Biotechnology, Santa Cruz, CA, USA
SOD2	Rabbit	Cell Signaling Technology, Inc., Danvers, MA, USA
β-actin	Mouse	Abcam, Cambridge, UK
IRDye 800CW	Anti-mouse	Li-Cor Biosciences, Bad Homburg, Germany
IRDye 800CW	Anti-rabbit	Li-Cor Biosciences, Bad Homburg, Germany
IRDye 680RD	Anti-mouse	Li-Cor Biosciences, Bad Homburg, Germany
IRDye 680RD	Anti-rabbit	Li-Cor Biosciences, Bad Homburg, Germany

## Data Availability

All the data generated or analyzed during this study are included in this article. This study includes no data deposited in external repositories. The data that support the findings of this study are available on request from the corresponding author.

## Supplementary Materials

The following supporting information can be downloaded at: https://www.mdpi.com/article/10.3390/cancers16193324/s1, Figure S1: ShcA family proteins; Supplementary Figure S2: Mitochondrial bioenergetics parameters in the MCF-7 clones.; Supplementary Figure S3: Mass spectrometry (MS)-proteomic analysis of the levels of individual subunits of the OXPHOS in MCF-7 clones; Supplementary Figure S4: Mass spectrometry (MS)-proteomic analysis of individual enzymes involved in the glycolysis determined in clones of the MCF-7 human breast cancer cell line.
