# Supplementary material for: p66Shc Protein—Oxidative Stress Sensor or Redox Enzyme: Its Potential Role in Mitochondrial Metabolism of Human Breast Cancer
Source: Cancers (Basel). 2024 Sep 28;16(19):3324. doi: 10.3390/cancers16193324 (PMC11476363; doi:10.3390/cancers16193324)
Supplement: Supplementary file 1 [file cancers-16-03324-s001.zip › cancers-3159761-supplementary.pdf]

Supplementary information

p66Shc protein – oxidative stress sensor or redox enzyme, its potential role in mitochondrial metabolism of human breast cancer

Monika Prill, Vilma A Sardão, Mateusz Sobczak, Dominika Nowis, Jedrzej Szymanski and Mariusz R. Wieckowski

Supplementary figures

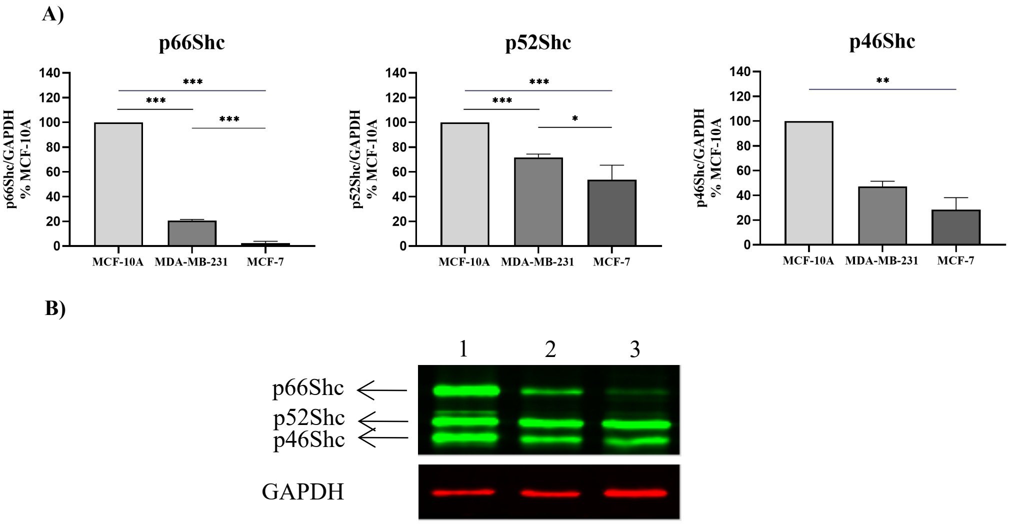

Legend: 1 – MCF-10A; **2** – MDA-MB-231; **3** – MCF-7

Supplementary Figure S1. ShcA family proteins. (A) Levels of individual isoforms of ShcA family proteins. (B) Representative result of the Western Blot analysis * $p<0.05$, ** $p<0.01$, *** $p<0.001$. The levels of individual proteins were standardized relative to the reference protein GAPDH. Legend: MCF-10A - non-tumorigenic normal mammary epithelial cell line; MDA-MB-231 and MCF-7 – human breast cancer cell lines.

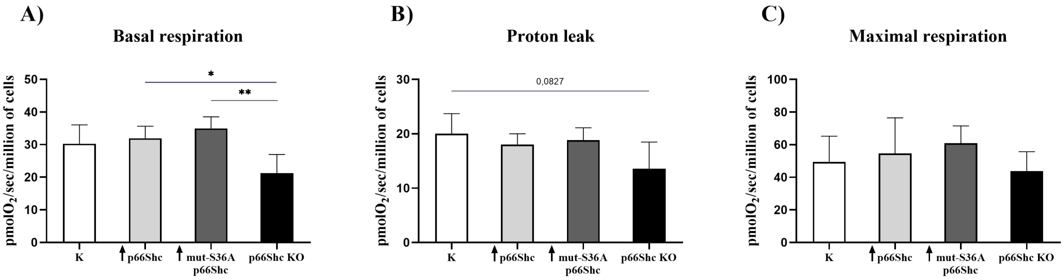

Supplementary Figure S2. Mitochondrial bioenergetics parameters in the MCF-7 clones. (A) Mitochondrial membrane potential and selected functional parameters of the mitochondrial respiratory chain: (B) Basal respiration, (C) Proton leak, (D) Maximal respiration.

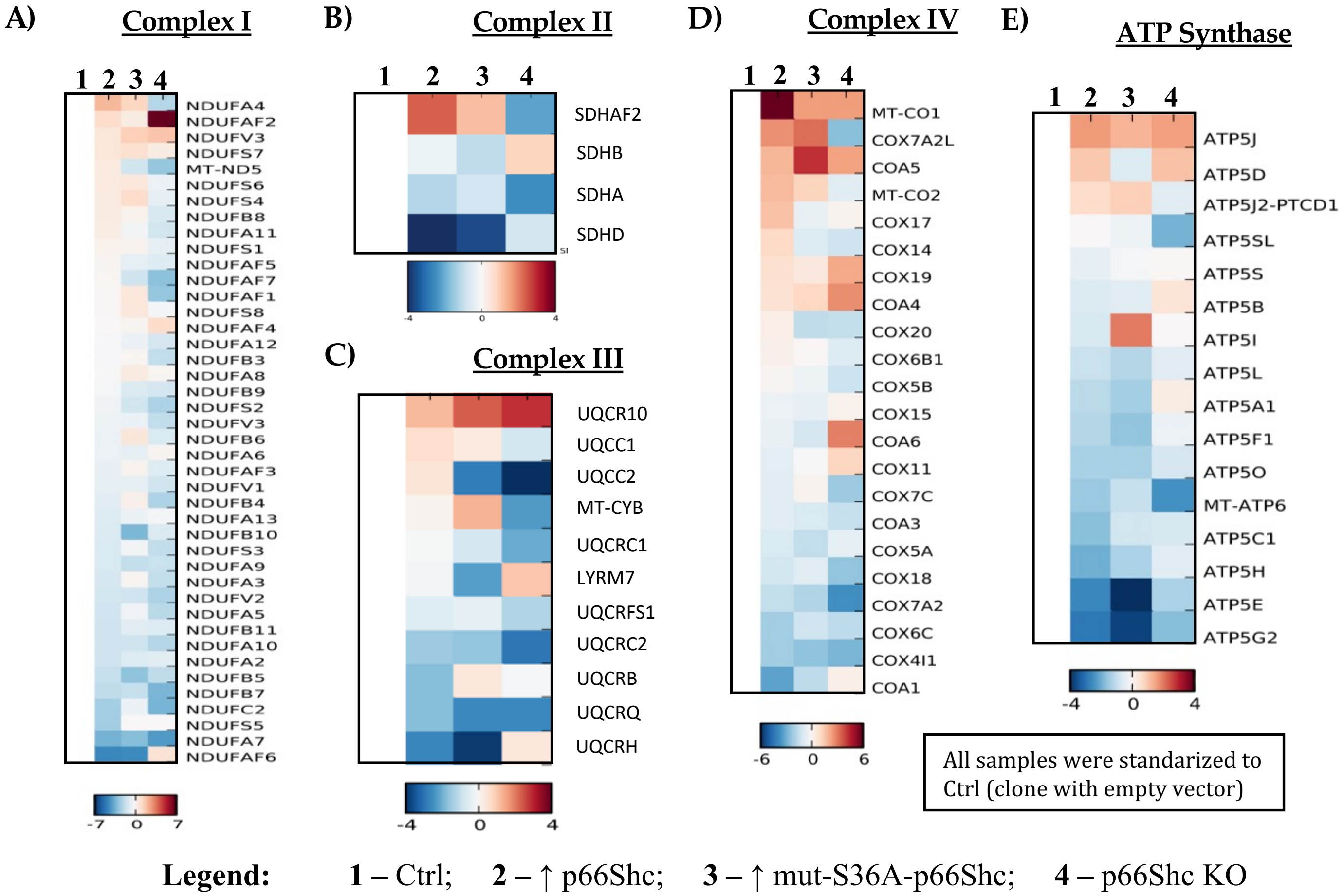

Supplementary Figure S3. Mass spectrometry (MS)-proteomic analysis of the levels of individual subunits of the OXPHOS in MCF-7 clones. (A) Complex I, (B) Complex II, (C) Complex III, (D) Complex IV, and (E) ATP synthase. Samples were standardized to the control clone (Ctrl). Blue color represents a decrease, while red color represents an increase of the protein levels.

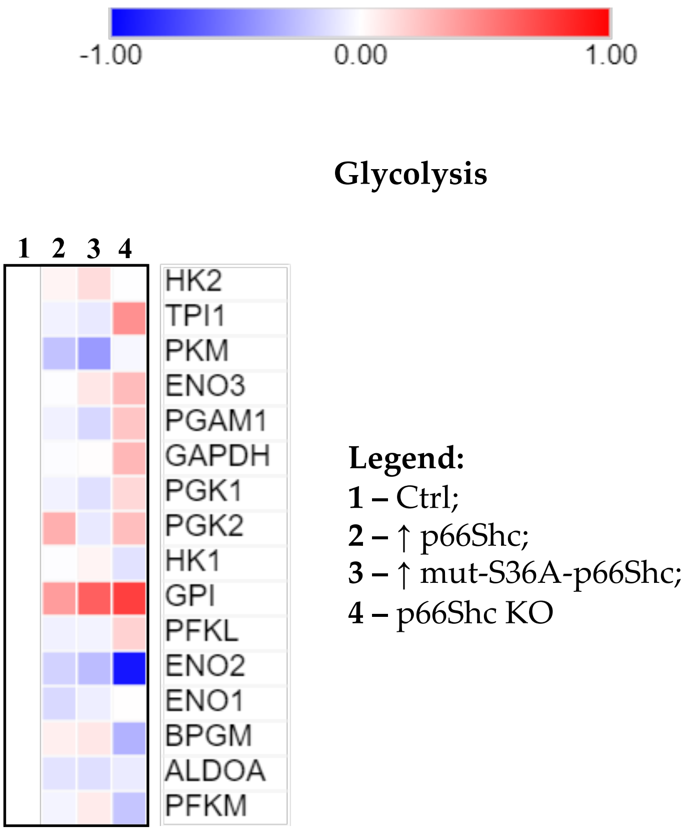

Supplementary Figure S4. Mass spectrometry (MS)-proteomic analysis of individual enzymes involved in the glycolysis determined in clones of the MCF-7 human breast cancer cell line. Blue color represents a decrease, while red color represents an increase of the protein levels.

Uncropped blots of Supplementary Figures

Uncropped, original image of Western blot presented in Supplementary data of the manuscript „p66Shc protein – oxidative stress sensor or redox enzyme, its potential role in mitochondrial metabolism of human breast cancer" are shown below.

Figure. 1B

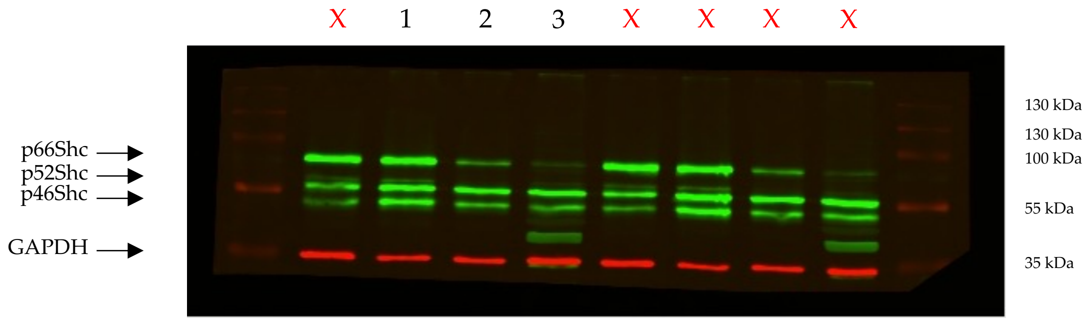

Legend:

X – Bands not included in manuscript
1 – MCF-10A
2 – MDA-MB-231
3 – MCF-7

Uncropped blots for figures included in the main version of the manuscript

Uncropped, original images of all Western blots presented in the manuscript „p66Shc protein – oxidative stress sensor or redox enzyme, its potential role in mitochondrial metabolism of human breast cancer" are shown below.

Figure. 2A

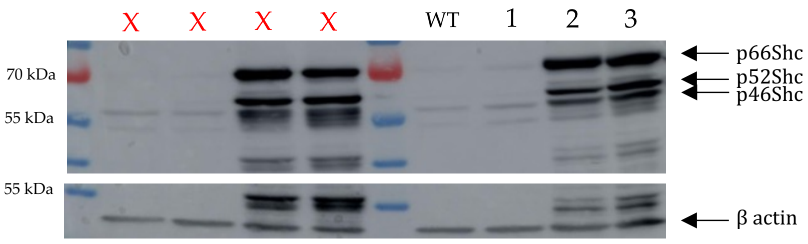

Legend:

X – Bands not included in manuscript
WT – Wild type of MDA-MB-231
1 – Ctrl
2 - ↑ p66Shc
3 - ↑ mut-S36A-p66Shc
4 - p66Shc KO

Figure. 2B

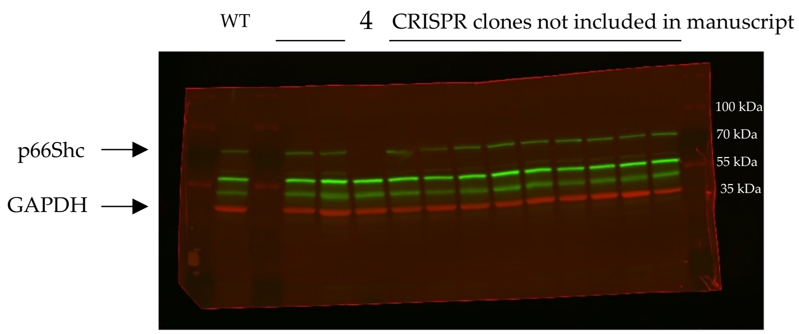

Figure. 3

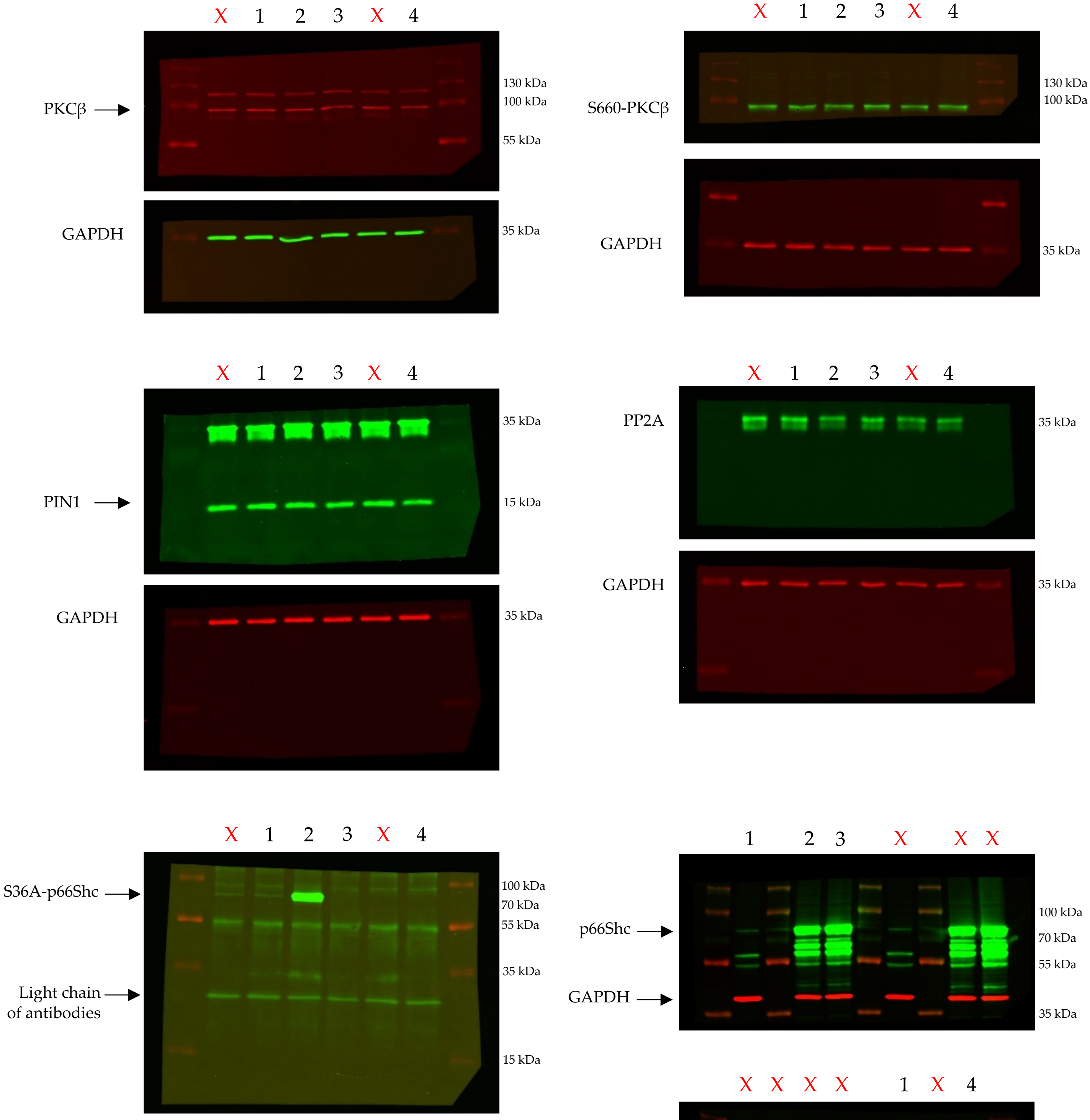

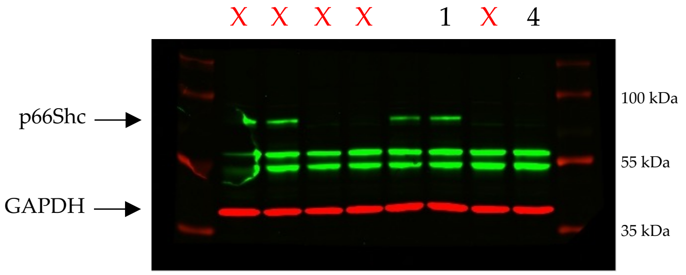

Legend:

X – Bands not included in manuscript
1 – Ctrl
2 - ↑ p66Shc
3 - ↑ mut-S36A-p66Shc
4 - p66Shc KO

<u>**Note:**</u> Due to the technical limitation of interferring the overexpressed p66Shc clones (↑p66Shc and ↑ mut-S36A-p66Shc) to neighbouring lane (p66Shc KO) during Western Blot procedure, we decided to perform analysis of p66Shc protein level in individual clones of MDA-MB-231 onto two separate gels. Each individual gel contained physically the same internal control sample (sample of Ctrl clone) that allowed us to later compare and merge the results from the individual blots.

Figure. 4B

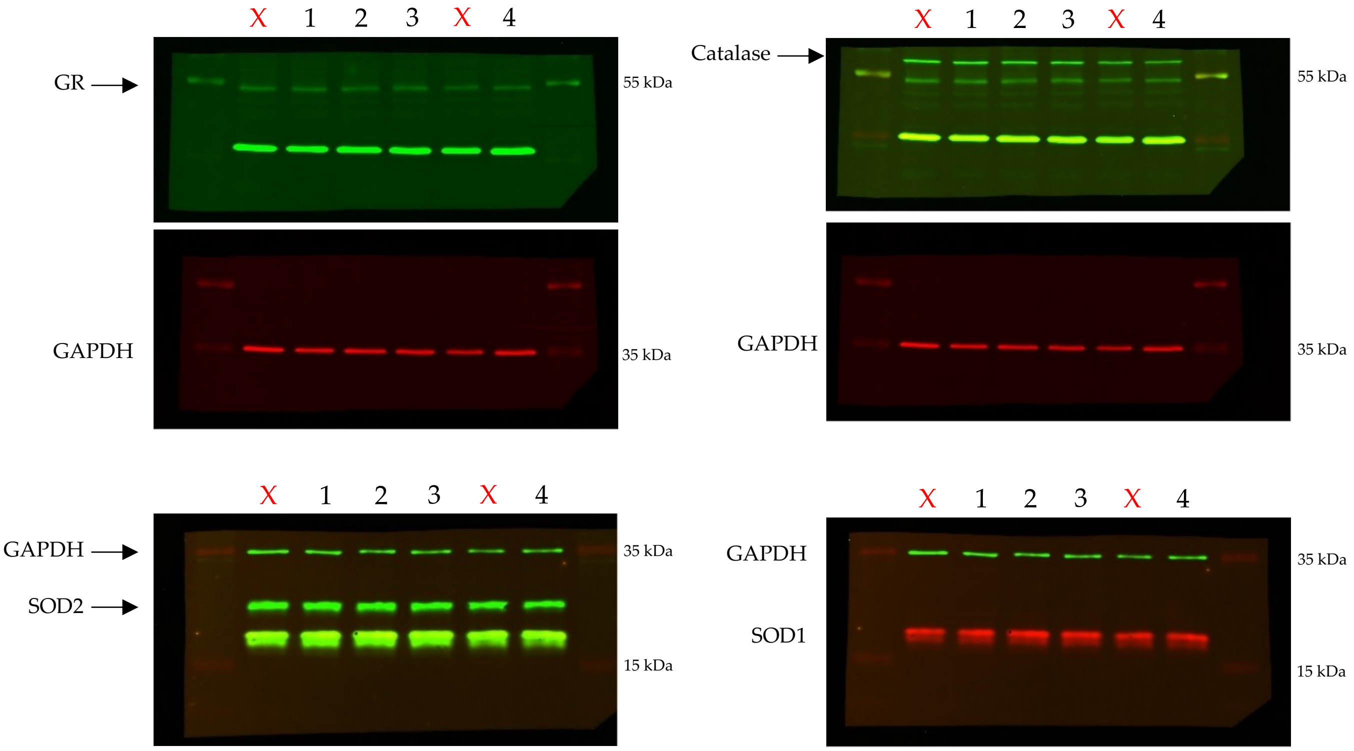

Legend:

X – Bands not included in manuscript
1 – Ctrl
2 - ↑ p66Shc
3 - ↑ mut-S36A-p66Shc
4 - p66Shc KO
